# A rheostat mechanism governs the bifurcation of carbon flux in mycobacteria

**DOI:** 10.1038/ncomms12527

**Published:** 2016-08-24

**Authors:** Paul Murima, Michael Zimmermann, Tarun Chopra, Florence Pojer, Giulia Fonti, Matteo Dal Peraro, Sylvie Alonso, Uwe Sauer, Kevin Pethe, John D. McKinney

**Affiliations:** 1 School of Life Sciences, Swiss Federal Institute of Technology in Lausanne (EPFL), CH-1015 Lausanne, Switzerland; 2 Institute of Molecular Systems Biology, Swiss Federal Institute of Technology in Zürich (ETHZ), CH-8093 Zürich, Switzerland; 3 Protein Crystallography Platform, Swiss Federal Institute of Technology in Lausanne (EPFL), CH-1015 Lausanne, Switzerland; 4 Department of Microbiology, Yong Loo Lin School of Medicine and Immunology Programme, Life Sciences Institute, National University of Singapore, Singapore 117456, Singapore; 5 Lee Kong Chian School of Medicine and School of Biological Sciences, Nanyang Technological University, 59 Nanyang Drive, Singapore 636 921, Singapore

## Abstract

Fatty acid metabolism is an important feature of the pathogenicity of *Mycobacterium tuberculosis* during infection. Consumption of fatty acids requires regulation of carbon flux bifurcation between the oxidative TCA cycle and the glyoxylate shunt. In *Escherichia coli*, flux bifurcation is regulated by phosphorylation-mediated inhibition of isocitrate dehydrogenase (ICD), a paradigmatic example of post-translational mechanisms governing metabolic fluxes. Here, we demonstrate that, in contrast to *E. coli*, carbon flux bifurcation in mycobacteria is regulated not by phosphorylation but through metabolic cross-activation of ICD by glyoxylate, which is produced by the glyoxylate shunt enzyme isocitrate lyase (ICL). This regulatory circuit maintains stable partitioning of fluxes, thus ensuring a balance between anaplerosis, energy production, and precursor biosynthesis. The rheostat-like mechanism of metabolite-mediated control of flux partitioning demonstrates the importance of allosteric regulation during metabolic steady-state. The sensitivity of this regulatory mechanism to perturbations presents a potentially attractive target for chemotherapy.

## Abstract (web summary)

Microbes survive in dynamic environments by modulating their intracellular metabolism. Here, the authors reveal that mycobacteria employ a rheostat-like mechanism to regulate carbon flux between the oxidative TCA cycle and the glyoxylate shunt during glucose-acetate diauxic shift.

## Graphical abstract

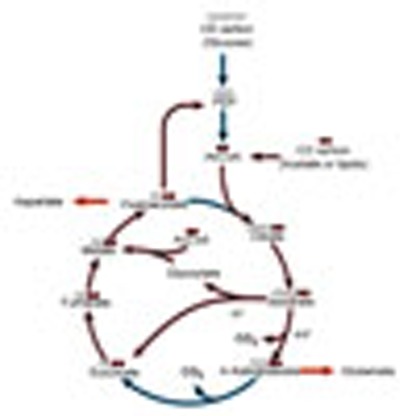

Tuberculosis (TB), caused by *Mycobacterium tuberculosis*, shares with HIV and malaria the distinction of being one of the deadliest infectious diseases of our time. Lipids and fatty acids are thought to serve as important sources of carbon and energy for *M. tuberculosis* during infection^1^,^2^. The dual use of these substrates places a premium on metabolic regulatory mechanisms to ensure a balance between metabolite oxidation for energy gain and metabolite conservation for biomass production.

Intermediates of the tricarboxylic acid (TCA) cycle that are diverted into biosynthetic pathways must be replenished by anaplerotic reactions. During growth on glycolytic substrates, anaplerosis involves transformation of glycolytic intermediates (C3-units) into TCA cycle intermediates (C4-units). These fluxes are absent during growth on fatty acids, which enter central carbon metabolism mainly as acetyl-CoA (C2-units). Instead, a fraction of the TCA cycle intermediate isocitrate is diverted into the glyoxylate shunt, bypassing the oxidative decarboxylation steps in the TCA cycle and replenishing intermediates that are used for biosynthesis of cellular constituents^3^. Since both pathways are essential under these conditions—the glyxoylate shunt for anaplerosis, the oxidative TCA cycle for energy and biosynthetic precursors—balancing the flux ratio at the bifurcation of these pathways is critical^4^. In enteric bacteria, the glyoxylate shunt is activated by transcriptional induction of the catabolite-repressed genes encoding isocitrate lyase (ICL) and malate synthase (MLS). After such transcriptional activation, the flux ratio between the oxidative TCA cycle and the glyoxylate shunt is controlled by post-translational regulation mediated by reversible phosphorylation^5^,^6^,^7^. This regulation is achieved by partial inactivation of isocitrate dehydrogenase (ICD), which competes with ICL for their shared substrate (isocitrate)^8^. The bifunctional enzyme AceK catalyzes both the phosphorylation and dephosphorylation of ICD to render the enzyme inactive and active, respectively^8^,^9^.

In contrast to *E. coli*, the molecular mechanisms that control partitioning of carbon fluxes between the TCA cycle and glyoxylate shunt are not understood in mycobacteria, despite the important role of flux bifurcation in *M. tuberculosis* pathogenicity^10^,^11^. Here, we report that phosphorylation of ICD does not play a role in controlling the bifurcation of isocitrate fluxes between the TCA cycle and glyoxylate shunt in *M. smegmatis*, which encodes only one ICD. In *M. tuberculosis* and *M. bovis* BCG, which encode two ICD isoforms^12^,^13^, we demonstrate that only ICD2 (homologue of ICD in *M. smegmatis*) has an important physiological role under the tested growth conditions. In the absence of ICD phosphorylation, we present evidence that flux partitioning between the TCA cycle and the glyoxylate shunt is mediated by cross-activation of ICD by glyoxylate produced by ICL.

## Results

### ICD2 is exclusively required for oxidative TCA cycle flux

**Figure 1:**
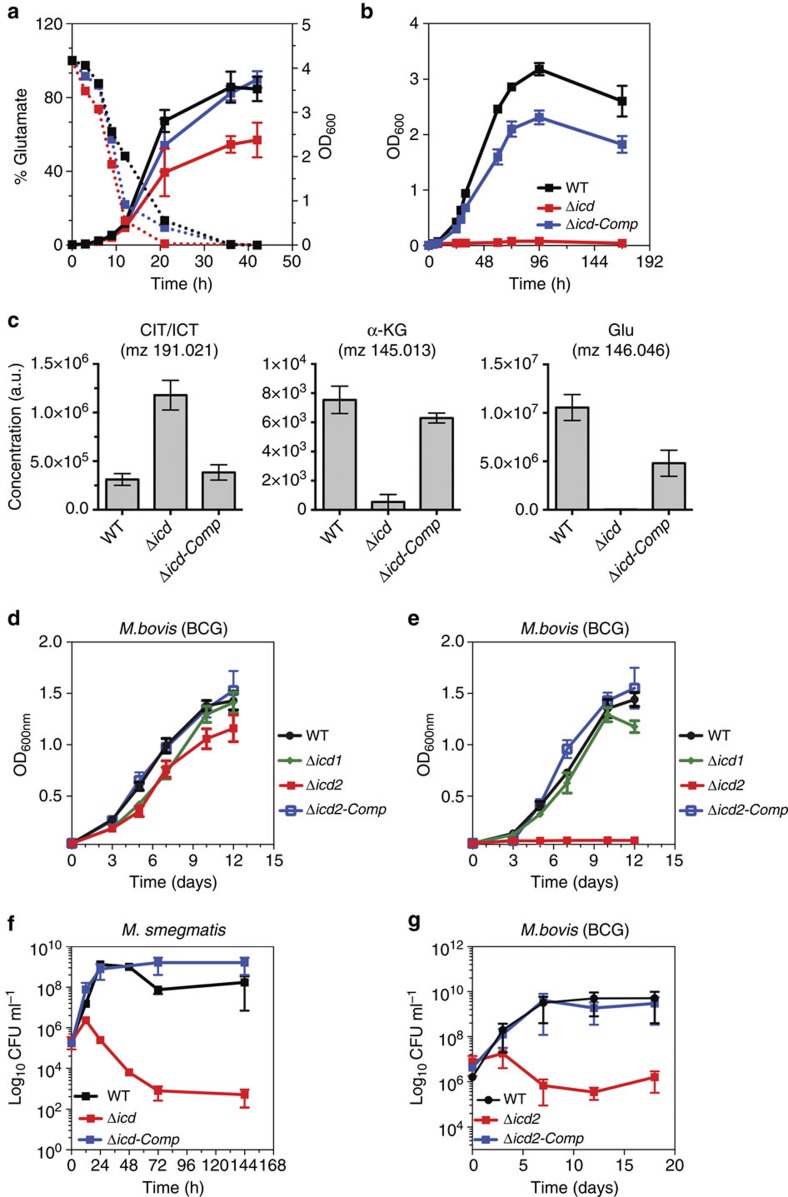
Loss of ICD2 results in glutamate auxotrophy and impaired viability. (**a**) Growth (OD_600_) of wild-type, Δ*icd* and Δ*icd attB*::P_np_*icd*-complemented strains of *M. smegmatis* in Middlebrook 7H9 medium. Solid lines, culture density (OD_600_). Dashed lines, glutamate concentration in culture medium. (**b**) Growth (OD_600_) of wild-type, Δ*icd* and Δ*icd attB*::P_np_*icd*-complemented strains of *M. smegmatis* in minimal medium supplemented with glucose and devoid of glutamate. Data are means±s.d. (*n*=3 independent experiments). (**c**) Intracellular metabolites in wild-type, Δ*icd* and Δ*icd attB*::P_np_*icd*-complemented strains of *M. smegmatis* 24 h after transferring cells into minimal medium supplemented with glucose and devoid of glutamate. Data are means±s.d. (*n*=3 independent experiments). CIT/ICT, citrate/isocitrate; α-KG, alpha-ketoglutarate; GLU, glutamate. (**d**,**e**) Growth (OD_600_) of wild-type, Δ*icd1*, Δ*icd2* and Δ*icd2 attB*::P_np_*icd2*-complemented strains of *M. bovis* BCG in minimal medium supplemented with glucose and glutamate (**d**) or without glutamate (**e**). (**f**) Survival (CFU) of wild-type, Δ*icd* and Δ*icd attB*::P_np_*icd*-complemented strains of *M. smegmatis* in minimal medium supplemented with glucose and devoid of glutamate. Data are means±s.d. (*n*=3 independent experiments each performed in triplicate). (**g**) Survival (CFU) of wild-type, Δ*icd2* and Δ*icd2 attB*::P_np_*icd2*-complemented strains of *M. bovis* BCG in minimal medium supplemented with glucose and devoid of glutamate. Data are means±s.d. (*n*=3 independent experiments each performed in triplicate).

*M. tuberculosis* and *M. bovis* BCG encode two distinct isoforms of ICD: ICD1 (409 AA) and ICD2 (745 AA). The *M. smegmatis* genome encodes a single ICD (743 AA), a homologue of ICD2 in *M. tuberculosis* and *M. bovis* BCG. Both isoenzymes (ICD1 and ICD2) are biochemically active *in vitro*^14^. To study the relative contributions of the isoenzymes to oxidative TCA cycle fluxes *in vivo*, we generated gene deletion strains. ICD activity is undetectable in ICD2-deficient *M. smegmatis* and ICD2-deficient *M. bovis* BCG but is indistinguishable from wild-type in ICD1-deficient *M. bovis* BCG (Table 1; Supplementary Table 1). An ICD2-deficient strain of *M. smegmatis* cultured in Middlebrook 7H9 medium shows a late growth phenotype that coincides with depletion of glutamate from the culture medium (Fig. 1a). Glutamate auxotrophy of the ICD2-deficient strain was confirmed by demonstrating that ICD2 is required for growth on minimal medium lacking glutamate (Fig. 1b; Supplementary Fig. 1). Incubation of ICD2-deficient bacteria in minimal medium without glutamate supplementation leads to decreased levels of metabolites downstream of ICD (α-ketoglutarate and glutamate) (Fig. 1c). In addition, loss of ICD2 increases the levels of metabolites upstream of ICD (citrate/isocitrate) compared with wild-type and complemented bacteria cultivated in media devoid of glutamic acid (Fig. 1c), as expected upon perturbation of a metabolic enzyme^15^. In *M. bovis* BCG, deletion of *icd2* results in glutamate auxotrophy, whereas deletion of *icd1* has no effect (Fig. 1d; Supplementary Table 1), and glutamate prototrophy is restored by complementation of the Δ*icd2* strain with a plasmid encoding *icd2* (Fig. 1e). ICD2-deficient *M. smegmatis* and *M. bovis* BCG lose viability over time when incubated in medium without glutamate supplementation, suggesting that energy metabolism or production of biosynthetic intermediates through the oxidative TCA cycle is essential for survival under these conditions (Fig. 1f,g).

We conclude that under the conditions tested ICD2 is responsible for carbon flux through the oxidative TCA cycle in both *M. smegmatis* and *M. bovis* BCG, whereas the physiological role of ICD1 in *M. bovis* BCG is currently unclear. Multiple attempts to delete the *icd2* gene in *M. tuberculosis* were unsuccessful, consistent with whole-genome mutagenesis studies suggesting that *icd2* might be essential in *M. tuberculosis*^16^.

### 3D structure of MsmICD does not support phospho-regulation

**Figure 2:**
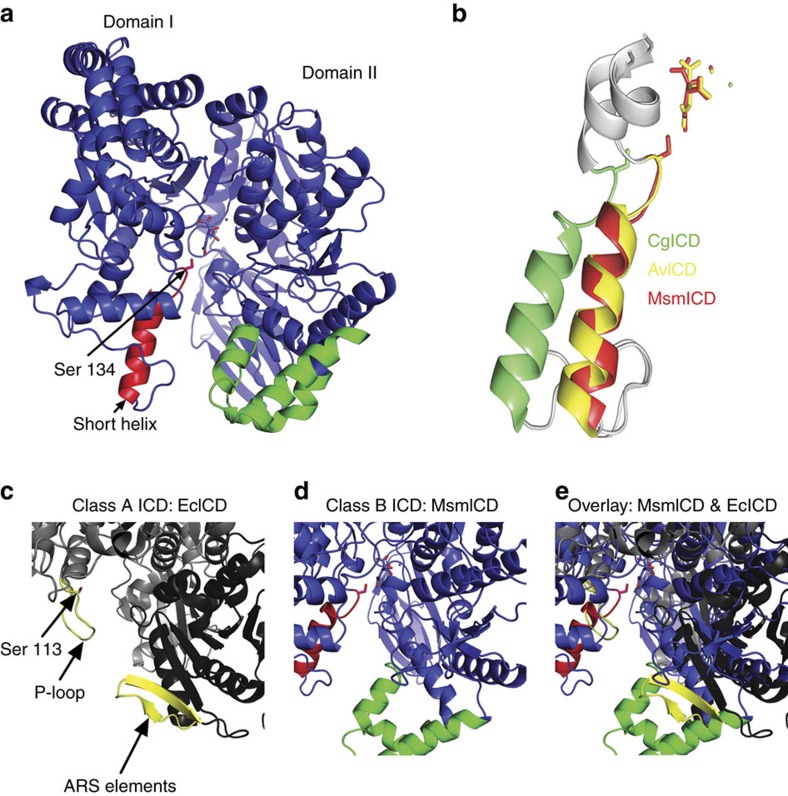
Three-dimensional structure of *M. smegmatis* ICD (MsmICD). (**a**) Schematic of two defined domains of MsmICD: a large domain (domain II) and a small domain (domain I). The corresponding ‘P-loop domain' is coloured in red, with Serine 134 depicted as a stick. The corresponding ‘ARS domain' is coloured in green. The substrate isocitrate and cofactor Mn2+ are depicted as sticks in the active site. (**b**) Overlay of the alpha-helical P-loops of MsmICD (red, this study), AvICD (yellow, PDB: 1ITW) and CgICD (green, PDB: 2B0T). (**c**) Class-A ICD of *E. coli*. Serine 113 extending from the flexible P-loop is depicted as a stick. (**d**) Class-B ICD of *M. smegmatis*. Unlike EcICD (PDB: 3LCB), which has a random coil P-loop and short ARS domain, MsmICD has a corresponding rigid alpha-helical structure and an elongated ‘ARS domain', which could sterically hinder the association of MsmICD with a regulatory kinase. (**e**) Overlay of EcICD and MsmICD illustrates the differences between the two proteins in the key regions that mediate interaction of EcICD with AceK.

A structural survey of bacterial ICDs revealed that only ICDs from Gram-negative bacteria are likely to be targets for the bifunctional kinase-phosphatase AceK^17^. However, by analogy to AceK, the mono-functional serine–threonine protein kinase G (PknG) in mycobacteria has been proposed to regulate fluxes through the TCA cycle by phosphorylation-mediated inactivation of ICD^18^,^19^. We used X-ray crystallography to solve the three-dimensional (3D) structure of *M. smegmatis* ICD (MsmICD) to evaluate whether it might support a phospho-regulatory mechanism. We determined the structure of MsmICD bound to isocitrate and manganese to a resolution of 2.8 Å using molecular replacement with ICD of *Azotobacter vinelandii* (AvICD) as template (Supplementary Table 2). MsmICD, the homologue of *M. tuberculosis* ICD2, is similar to the NADP^+^-dependent ICD of *Corynebacterium glutamicum* (CgICD) with a sequence identity of 59% and an rmsd of 3.9 Å of all C-alpha atoms (DaliLite v.3). The tertiary structure of MsmICD (PDB: 4ZDA) consists of two distinct domains, similar to the ICD structures of AvICD and CgICD (Fig. 2a). A cleft region between the two domains contains the active site, as described for other ICD enzymes^20^,^21^.

The phospho-regulatory P-loop of *E. coli* ICD (EcICD), comprising amino acid residues 94–113, includes a glycine-rich motif forming a highly flexible structure (Fig. 2c). In contrast, the equivalent amino acid residues in MsmICD are more structured, forming a short α-helix (amino acid residues 119–134) (Fig. 2d) similar to the short α-helix loop in CgICD and AvICD (Fig. 2b)^20^,^21^. Overlaid with EcICD, this short α-helix limits access to the active site and would sterically hinder binding of AceK to the AceK recognition sequence (ARS) (Fig. 2e). In contrast to the EcICD ARS elements, which form a β-sheet (Fig. 2c), the corresponding structural elements of MsmICD resemble a Class-B ICD (Fig. 2d)^17^. Structural comparison of representative ICD structures from enterobacteria that encode an AceK homologue reveals remarkable sequence conservation in their ARS elements compared with MsmICD and CgICD (Supplementary Fig. 2). Indeed an AceK–EcICD complex structure illustrates a highly specific recognition and intimate interaction between the enzyme and the kinase, similar to several documented cases in eukaryotic protein kinases^9^. The discontinuous regions on EcICD that interact with AceK are: the P-loop from domain (I) and the twisted antiparallel beta-sheet (ARS) from domain (II). Notably, modification of these recognition sites on EcICD, analogous to the amino acid residue differences in MsmICD, significantly impairs the ability of AceK to interact with EcICD^9^,^22^. Consistent with the genetic and biochemical evidence presented hereafter, the structure of MsmICD argues against a phosphorylation-mediated mechanism for ICD regulation in mycobacteria.

### PknG kinase does not regulate ICD activity

**Figure 3:**
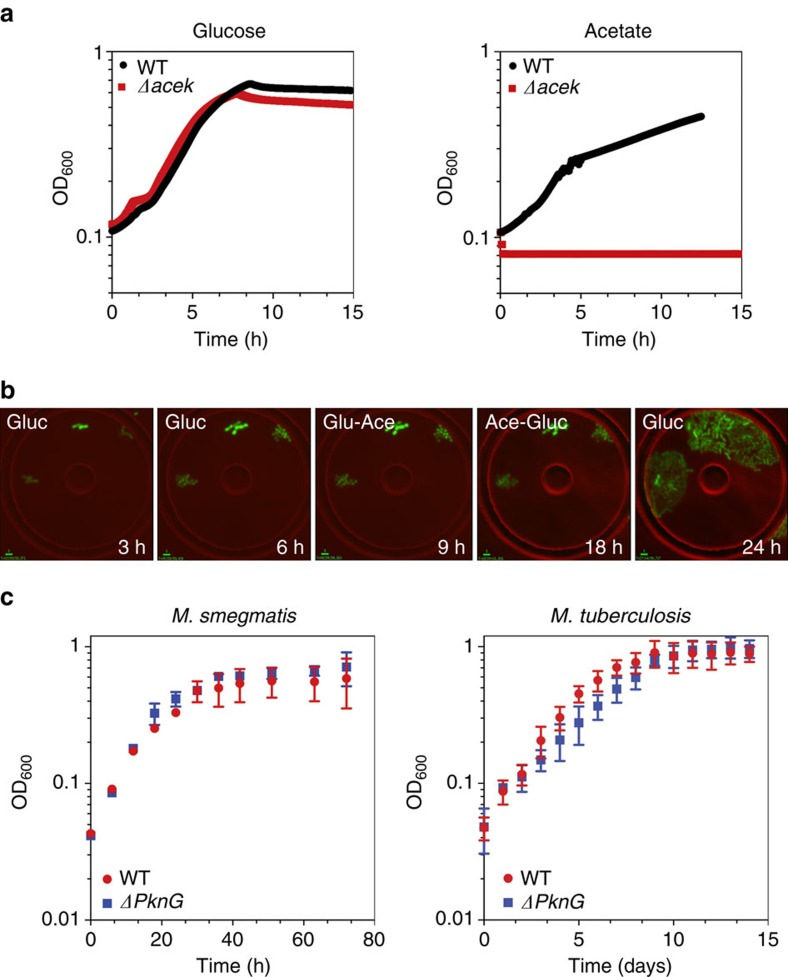
Phosphorylation-mediated branch-point regulation. (**a**) Growth (OD_600_) of wild-type and Δ*aceK* strains of *E. coli* on minimal medium containing glucose (left) or acetate (right). Data are means±s.d. (*n*=3 independent experiments). (**b**) Time-lapse series of GFP-expressing *E. coli* Δ*aceK* cells grown in a microfluidic device and imaged on phase-contrast and fluorescence channels (merged) at 5-minute intervals. Flow medium was M9 minimal medium containing glucose (0–6 h) before switching to acetate (6–18 h) and then back to glucose (18–24 h). Scale bar, 3 μm. (**c**) Growth (OD_600_) of wild-type and Δ*pknG* strains of *M. smegmatis* (left) or *M. tuberculosis* (right) on minimal medium containing acetate. Data are means±s.d. (*n*=3 independent experiments).

To further clarify whether PknG contributes to the regulation of ICD activity, we constructed PknG null strains and measured their growth kinetics and ICD specific activity in carbon-defined media. Although deletion of AceK increases ICD activity by several-fold in *E. coli*, PknG deficiency has no impact on ICD activity in mycobacteria (Table 2). Furthermore, we found that PknG is dispensable for growth of mycobacteria on acetate (Fig. 3c; Supplementary Fig. 3), whereas AceK is essential for growth of *E. coli* on acetate (Fig. 3a,b; Supplementary Movie 1). These observations suggest that PknG is not involved in modulating ICD activity during growth of mycobacteria on acetate. This conclusion is further supported by the fact that immunoprecipitated ICD fractions from glucose-grown and acetate-grown cells show indistinguishable specific activity (0.85±0.21 and 0.87±0.18 U μg^−1^, respectively).

### Bifurcation of fluxes at the TCA cycle branch-point

**Figure 4:**
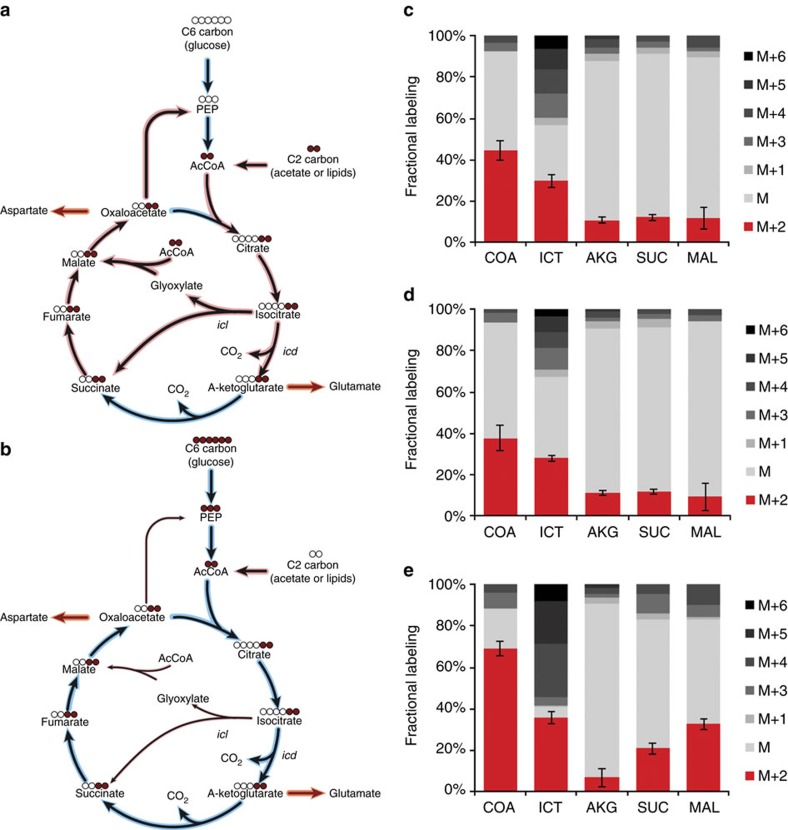
Bifurcation of metabolic fluxes. (**a**) Schematic of fluxes into the TCA cycle and glyoxylate shunt following shift from glucose (blue shading) to radiolabelled acetate (red shading). (**b**) Schematic of fluxes into the TCA cycle and glyoxylate shunt following shift from acetate (red shading) to radiolabelled glucose (blue shading). (**c**,**d**) ^13^C-labelling of metabolites in wild-type (**c**) and Δ*icl1* Δ*icl2* (**d**) *M. smegmatis* 320 s after shifting glucose to [U-^13^C]-glucose. Comparison of the M+2 isomers of isocitrate, α-ketoglutarate and succinate demonstrate absence of carbon flux through the glyoxylate shunt during growth on glucose. (**e**) ^13^C-labelling of metabolites in wild-type *M. smegmatis* 80 s after shifting from acetate to [U-^13^C]-acetate illustrate carbon flux bifurcation to the glyoxylate shunt and the oxidative TCA cycle.

Bacteria growing on C2-carbon sources use the glyoxylate shunt for anaplerosis of the TCA cycle while maintaining oxidative TCA cycle activity for the production of energy and α-ketoglutarate-related biomass precursors. In the absence of phosphorylation-mediated modulation of ICD activity, we evaluated whether partitioning of fluxes between the TCA cycle and the glyoxylate shunt occurs in mycobacteria. We used dynamic ^13^C-tracing to estimate the flux ratio between the two competing pathways^23^. The conversion of isocitrate to succinate is either through the oxidative branch of the TCA cycle or through the glyoxylate shunt. Since both pathways conserve the two carbon atoms of acetyl-CoA, the initial dynamics of the M+2 mass isomer of succinate (before extensive reshuffling of labelled carbons) after shifting cells from unlabelled acetate to [U-^13^C]-acetate are informative to determine the relative contributions of the two converging metabolic fluxes (Fig. 4a,b). Shifting cells from unlabelled glucose to [U-^13^C]-glucose resulted in similar dynamics of the M+2 isomer fraction of α-ketoglutarate and succinate over the first 320 s that were comparable between wild-type (Fig. 4c) and ICL-deficient (Δ*icl1* Δ*icl2*) (Fig. 4d) strains. These results demonstrate that fluxes through the glyoxylate shunt are negligible in glucose-grown cells. In contrast, shifting acetate-grown cells to [U-^13^C]-acetate resulted in a twofold faster M+2 isomer accumulation for succinate compared with α-ketoglutarate during the first 80 s, which demonstrates substantial flux through the glyoxylate shunt (Fig. 4e). Isotopomer balancing, assuming pseudo-steady-state labelling after 80 s, indicated that 32–38% of the succinate molecules are generated through the glyoxylate shunt and 62–68% are generated through the oxidative TCA cycle during growth on acetate. This is comparable to the determined 1:2 to 1:3 ratio of fluxes between the glyoxylate shunt and the oxidative TCA cycle for acetate-grown *E. coli* and *C. glutamicum*, respectively 4,24. Thus, the emerging picture of mycobacterial metabolism is of exclusive utilization of the TCA cycle during growth on glucose and bifurcation of fluxes between the TCA cycle and glyoxylate shunt during growth on acetate.

### Mycobacteria do not inactivate ICD during growth on acetate

In enterobacteria, the split-ratio of fluxes at the bifurcation of the oxidative TCA cycle and glyoxylate shunt is maintained by partial inactivation of ICD, which is reversibly phosphorylated by the bifunctional kinase-phosphatase AceK^8^. Although phosphorylation-mediated inhibition of ICD does not seem to play a role in mycobacteria (see above), we investigated whether other mechanisms might contribute to ICD regulation by measuring total ICD enzyme activity in cells grown on glucose or acetate as the sole source of carbon and energy. Consistent with previous studies, we found that total ICD activity in *E. coli* cell extracts is threefold lower in acetate-grown cells compared with glucose-grown cells. In sharp contrast, we found that total ICD activity in mycobacterial cell extracts is increased rather than decreased in acetate-grown cells compared with glucose-grown cells in all of the species that we tested (*M. smegmatis*, *M. bovis* BCG and *M. tuberculosis*) (Tables 1 and 2). These observations confirm that mycobacteria do not use phosphorylation-mediated inhibition of ICD to balance flux partitioning between the TCA cycle and the glyoxylate shunt.

### ICD2 expression is independent of the carbon source

Since ICD2 seems to be solely responsible for intracellular ICD activity and carbon flux through the oxidative TCA cycle under standard growth conditions *in vitro*, we focused on this isoenzyme to investigate the mechanism of flux partitioning between the TCA cycle and the glyoxylate shunt. We found that increased ICD activity in acetate-grown cells (Table 1) cannot be attributed to changes in ICD2 protein abundance, which is similar in cells grown on glucose or acetate (Supplementary Fig. 4). Stable post-translational modifications also cannot account for increased ICD activity in acetate-grown cells, given that ICD specific activity is similar in immunoprecipitated fractions from cells grown on glucose. These results suggest that increased ICD activity in acetate-grown cells cannot be explained by changes in the intrinsic activity of ICD2 alone and might involve additional carbon-responsive elements. ICL is a putative candidate because it competes with ICD for a shared substrate (isocitrate) and ICL expression is induced in cells grown on acetate compared with glucose (Table 3; Supplementary Fig. 4a).

### Metabolic cross-activation of ICD by ICL

**Figure 5:**
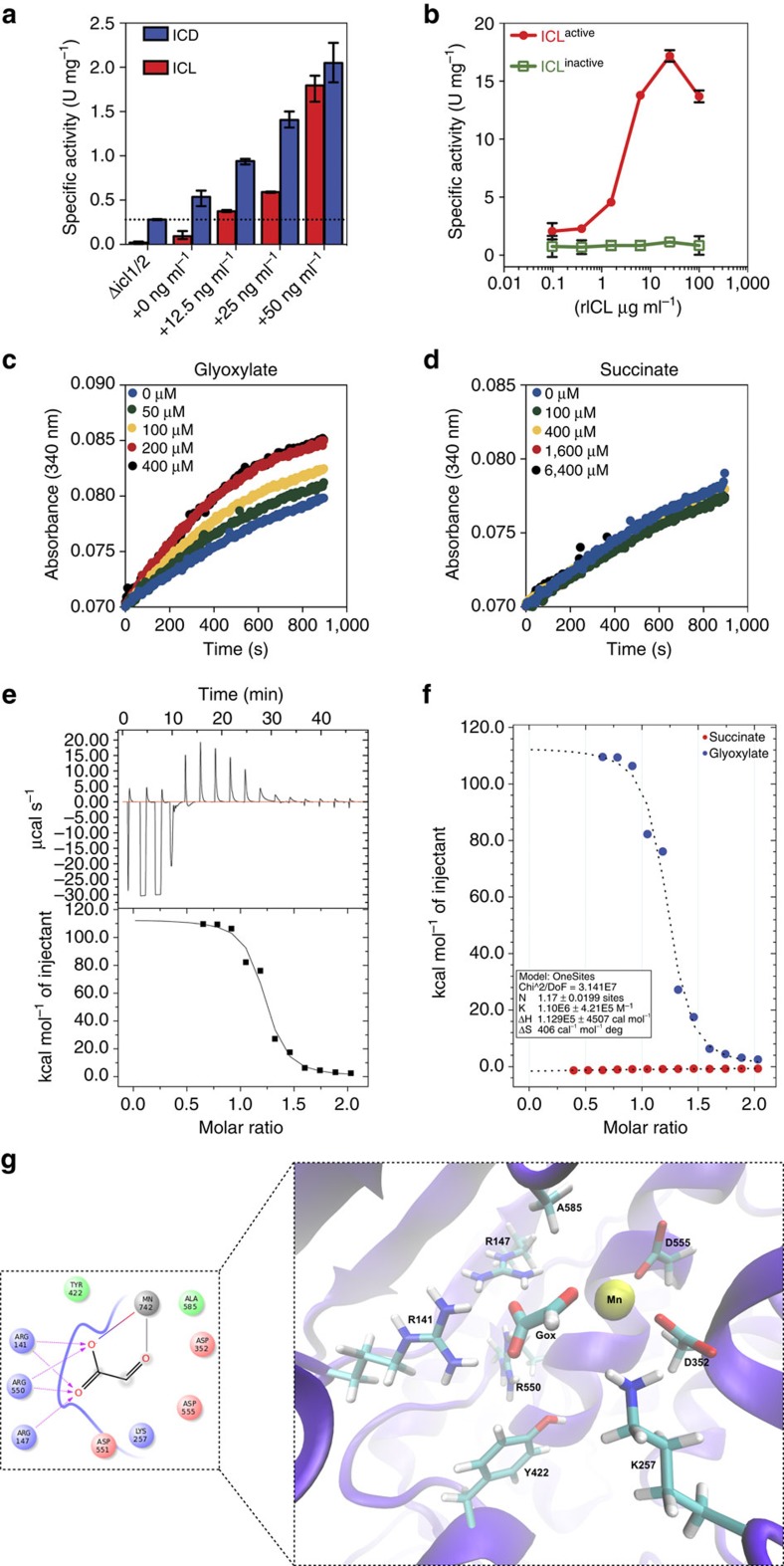
ICD is activated by glyoxylate but not succinate. (**a**) ICD activity *in vivo* increases concomitantly with ICL1 activity in a strain of *M. smegmatis* that conditionally expresses *icl1* from an ATc-inducible promoter. Cells were induced with the indicated concentration of ATc for 18 h before measurement of ICD and ICL1 activities in cell-free extracts. Data are means±s.d. (*n*=3 independent experiments). (**b**) rICD activity *in vitro* is stimulated by active rICL1 in a dose-dependent manner. A catalytically inactive mutant of rICL1 (rICL1^KKAGA^) does not stimulate ICD activity. (**c**) rICD activity *in vitro* is stimulated by glyoxylate in a dose-dependent manner. (**d**) rICD activity *in vitro* is not stimulated by succinate. (**e**) Competitive ITC trace of a solution of glyoxylate-MsmICD binding in 10 mM Tris (pH 7.5), 10 mM EDTA, 50 mM NaCl_2_ and 2.5 mM MgCl_2_. Glyoxylate (150 mM) was titrated into MsmICD (15 mM) in the same buffer. Data are representative of at least three independent experiments. (**f**) Kinetics of glyoxylate and succinate interaction with MsmICD. Data was fitted into a single-site binding equation. (**g**) Detailed view of putative glyoxylate binding site close to the protein active site. Interacting residues Arg147, Arg550, Arg141, Tyr422, Ala585, Asp352, Asp555, Lys257 and Asp551 are shown in stick representation and the magnesium (Mn) as a yellow sphere. (Insert: detailed view of MsmICD–glyoxylate interaction.)

We used a strain of *M. smegmatis* with *icl1* transcribed from an anhydrotetracycline (ATc)-inducible promoter (Supplementary Fig. 5) to demonstrate that increased expression of ICL1 is accompanied by increased ICD activity in a dose-dependent manner (Fig. 5a). These results suggest that ICL1 cross-activates ICD2 *in vivo* through a direct or indirect mechanism. We reconstituted this effect *in vitro* using recombinant enzymes. Consistent with the *in vivo* data (Fig. 5a), we found that rICD2 activity increases in proportion to the amount of rICL1 added to the reaction mix (Fig. 5b). Cross-activation is dependent on ICL enzymatic activity, because an inactive catalytic-site variant of ICL1 (rICL1^KKAGA^) has no rICD2-stimulatory activity *in vitro* (Fig. 5b; Supplementary Fig. 6).

These observations suggest that ICL-dependent stimulation of ICD2 activity might be mediated by metabolic cross-activation rather than direct protein–protein interaction. Consistent with this hypothesis, we found that rICD2 activity *in vitro* is stimulated by direct addition of glyoxylate to the reaction mix (Fig. 5c), whereas addition of succinate has no effect (Fig. 5d). Stimulation of ICD activity by glyoxylate is dose-dependent up to a concentration of 200 μM, consistent with the intracellular concentration of glyoxylate that we measured in acetate-grown cells (∼20 μM) and about fourfold lower in glucose-grown cells, which is similar to data from *E. coli* cultures^25^. Indeed, these measurements are likely to underestimate the true concentrations of intracellular glyoxylate due to the inherent instability and reactivity of this compound. Moreover, it is likely that glyoxylate concentrations fluctuate over time and individual cells may transiently contain higher levels than the measured population average^26^,^27^,^28^, consistent with the rheostat-like mechanism of metabolite-mediated feedback regulation proposed here.

Finally, we employed isothermal titration calorimetry (ITC) to measure the binding affinity of glyoxylate for MsmICD (Fig. 5e). Thermodynamic parameters revealed that glyoxylate binds to MsmICD with high affinity and a stoichiometry of 1:1 (Table 4). Glyoxylate contributed favourably to the binding entropy (Δ*S*=406 cal mol^−1^), suggesting the presence of conformational changes. Importantly, MsmICD binding was not detected for succinate, consistent with the absence of ICD-stimulatory activity *in vitro* (Fig. 5f). To investigate the mechanism of glyoxylate-mediated activation of ICD, we attempted to co-crystallize apo-MsmICD with glyoxylate but we were unable to resolve glyoxylate in the resulting crystal structures. Therefore, we used the MsmICD crystal structure to perform computational docking studies with glyoxylate to generate models of the MsmICD–glyoxylate complex structure. The docking studies suggest that glyoxylate binds close to the active site of MsmICD, making key interactions with amino acid residues around the catalytic site (Fig. 5g; Supplementary Fig. 7).

## Discussion

**Figure 6:**
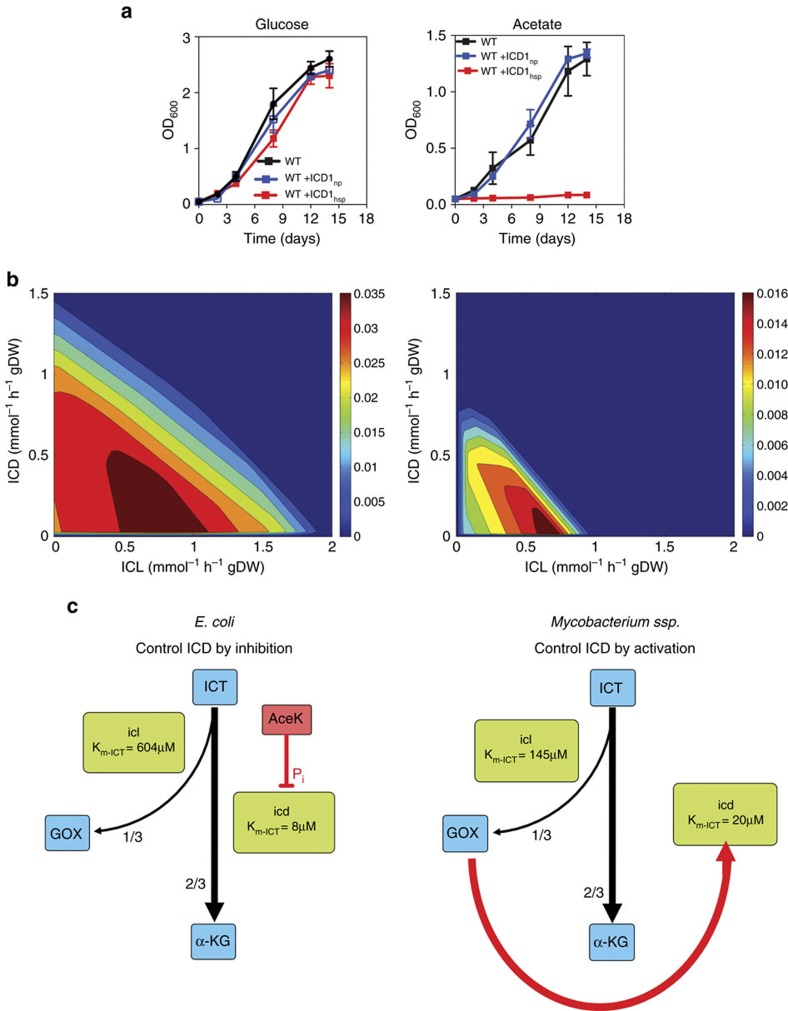
Dysregulation of the ICL/ICD rheostat balance impairs growth on acetate. (**a**) Growth of wild-type, *attB*::P_np_icd1 and *attB*::P_hsp_icd1 strains of *M. bovis* BCG in glucose-based (left) or acetate-based (right) minimal medium without glutamate. The *attB*::P_np_icd1 strain expresses *icd1* from the native promoter. The *attB*::P_hsp_icd1 strain overexpresses *icd1* from the strong *hsp60* promoter. Data are means±s.d. (*n*=3 independent experiments). (**b**) Computationally modelled phase planes of carbon flux between ICD and ICL in glucose-based (left) or acetate-based (right) medium. Heat maps show scales of predicted growth (maroon, best growth; blue, zero growth) at varying levels of ICD/ICL flux. The dynamic range of ICD flux allowing optimal growth at each ICL flux value is narrower in acetate-grown cells compared with glucose-grown cells. (**c**) Bifurcation of metabolic fluxes between the oxidative TCA cycle and the glyoxylate shunt in *E. coli* and mycobacteria. In *E. coli*, the substrate *K*_m_ of ICL is almost 100-fold higher than the substrate *K*_m_ of ICD. Partial inhibition of ICD by AceK-mediated phosphorylation enables partitioning of one-third of the flux into the glyoxylate shunt with the remaining two-thirds of the flux continuing through the oxidative TCA cycle. In mycobacteria, the substrate *K*_m_ of ICL1 is only slightly higher than the substrate *K*_m_ of ICD2. In this context, glyoxylate-mediated activation of ICD functions as a metabolic rheostat to maintain the optimal partitioning of fluxes between the TCA cycle and the glyoxylate shunt. ICT, isocitrate; GOX, glyoxylate; α-KG, alpha-ketoglutarate.

Despite the importance of fatty acid catabolism during *M. tuberculosis* infection^10^, the mechanisms that regulate the partitioning of metabolite fluxes at the bifurcation of the oxidative TCA cycle and the glyoxylate shunt remain unknown. Strict regulation of fluxes at this metabolic branch-point is essential during catabolism of C2-units derived from fatty acids in order to balance anaplerotic fluxes (glyoxylate shunt) and fluxes generating energy and biosynthetic precursors (TCA cycle) (Supplementary Fig. 8). Here, we demonstrate that this balance is mediated by metabolite-based regulation of ICD2 activity, whereas imbalancing of branch-point fluxes by overexpression of ICD1 inhibits growth on acetate (Fig. 6a; Supplementary Table 3). This growth-inhibitory effect is presumably caused by directing too much flux into the oxidative TCA cycle at the expense of the glyoxylate shunt, analogous to *E. coli* mutants deficient in phosphorylation-mediated inhibition of ICD^29^. This interpretation is consistent with limited viable flux combinations of ICL and ICD on acetate compared with glucose, as modelled by computational flux-balance analysis using either of these two substrates as the sole carbon source (Fig. 6b). Loss of ICD activity not only impairs growth on media with acetate as the sole carbon source but also leads to the loss of viability over time. These observations suggest that perturbation of flux partitioning at the branch-point of the TCA cycle and glyoxylate shunt might present a potential strategy for chemotherapy. This could be achieved by modulating the metabolic fluxes to favour the TCA cycle over the glyoxylate shunt, which would impair anaplerosis in cells catabolizing fatty acid substrates during infection *in vivo*^30^.

The architecture of the regulatory circuit that controls bifurcation of fluxes at the branch-point of the TCA cycle and glyoxylate shunt is strikingly different in mycobacteria compared with the established regulatory paradigm in *E. coli*. In *E. coli*, branch-point partitioning is controlled by phosphorylation-mediated inactivation of ICD, which diverts flux away from the TCA cycle and into the glyoxylate shunt (Fig. 6c)^8^. In line with the reported architecture of mycobacterial ICD1 (ref. 31), we find that the 3D architecture of ICD2 appears incompatible with phosphorylation-mediated control of enzymatic activity. It is perhaps not surprising, therefore, that all known and putative AceK proteins exist only in Gram-negative bacteria. Correspondingly, ICD proteins from the more distantly related Actinobacteria, including mycobacteria, seem to have lost (or perhaps never had) these AceK-interacting elements.

Here, we show that branch-point partitioning in mycobacteria is controlled by metabolite-mediated activation of ICD, which diverts flux away from the glyoxylate shunt and into the TCA cycle. Cross-activation of ICD is mediated by glyoxylate, which is ideally suited for this regulatory role because it is a unique product of the ICL-catalyzed cleavage of isocitrate to glyoxylate and succinate. According to this scheme, if glyoxylate levels rise, the corresponding increase in ICD activity ensures that carbon flux will shift towards the TCA cycle, leading to a decline in glyoxylate levels. Conversely, if glyoxylate levels fall, the corresponding decrease in ICD activity ensures that carbon flux will shift towards the glyoxylate shunt, leading to a recovery of glyoxylate levels. Thus, we propose that glyoxylate-mediated activation of ICD functions as a molecular rheostat to maintain the proper balance of fluxes between the TCA cycle and the glyoxylate shunt under steady-state conditions (Fig. 6c). In computational simulations, a similar partitioning of fluxes could be achieved by increasing the activity of ICL above a threshold in *E. coli* devoid of AceK^32^, suggesting that ICL can govern flux partitioning in the absence of phosphorylation-mediated inactivation of ICD.

Competitive displacement isothermal titration calorimetry revealed the true potency of glyoxylate, providing a *K*_A_ of 1.129e^5^M^−1^ that is driven by a positive endothermic binding entropy (Δ*S*=406 cal mol^−1^). Computational simulations of glyoxylate interaction with ICD suggest that the molecule binds to the enzyme at a site close the catalytic site. Characterization of this putative allosteric interaction and localization of the binding site of glyoxylate could provide new opportunities for therapeutic intervention, as flux partitioning between the TCA cycle and glyoxylate shunt is critical for survival of *M. tuberculosis* during infection. Similar to other NADP^+^-dependent ICDs, mycobacterial ICD2 forms dimers in solution^14^,^33^. According to the ultrasensitivity theory^34^, such multimeric structures can mediate cooperative binding of substrates or allosteric effector molecules, thus enabling rapid and switch-like responses to environmental changes^35^.

The rheostat-like mechanism proposed here involves metabolite-mediated enzyme activation to achieve a balanced bifurcation of fluxes between two pathways, whereas in *E. coli* the same flux balance is achieved by phosphorylation-mediated enzyme inhibition. In future it will be important to determine whether a rheostat-like mechanism can be generalized to other bacterial species in which ICD activity is not repressed during growth on C2-carbon substrates^36^,^37^,^38^,^39^. Although metabolite-mediated control has been implicated in metabolic adaptation during switching between distinct nutritional environments^40^,^41^,^42^, the results presented here also demonstrate its importance for metabolic homoeostasis under conditions of steady-state growth.

## Methods

### Bacterial strains and culture conditions

*E. coli* K-12 MG1655 was the reference wild-type strain used in all *E. coli* experiments. The Δ*aceK* null *E. coli* strain was obtained from the Yale *E. coli* Genetic Stock Center (CGSC#10859). Yellow fluorescent protein (YFP)-expressing derivatives of both *E. coli* strains were made by transformation with a plasmid encoding YFP^43^. *M. tuberculosis* Erdman, *M. tuberculosis* H37Rv, *M. tuberculosis* CDC1551, *M. tuberculosis* HN787, *M. bovis* BCG (Pasteur 1173P2), *M. smegmatis* mc^2^155 and derivative strains (Supplementary Table 4) were grown in Middlebrook 7H9 liquid medium (Difco) supplemented with 0.5% albumin, 0.2% glucose, 0.085% NaCl, 0.5% glycerol and 0.05% tyloxapol. Cultures were grown at 37 °C to mid-log phase, corresponding to optical density at 600 nm (OD_600_) of ∼0.5. Aliquots were stored in 15% glycerol at −80 °C and thawed at room temperature before use; individual aliquots were used once and discarded. When required, the medium was supplemented with 75 μg ml^−1^ hygromycin (Roche Diagnostics) or 25 μg ml^−1^ kanamycin (Sigma-K4378). *E. coli* MG1655 and derivative strains (Supplementary Table 4) were grown in LB liquid medium (Difco). When required, the medium was supplemented with 25 μg ml^−1^ kanamycin, 25 μg ml^−1^ chloramphenicol (Sigma-C0378) or 100 μg ml^−1^ ampicillin (Sigma-A0166).

For growth with single carbon sources, bacteria were cultured in M9 minimal medium (Difco) supplemented with 2 g l^−1^ of glucose or acetate, or in defined medium containing 2.5 g l^−1^ Na_2_HPO_4_, 1 g l^−1^ KH_2_PO_4_, 0.5 g l^−1^ glutamic acid, 1 mg l^−1^ pyridoxine, 0.5 mg l^−1^ biotin, 15 mg l^−1^ ferric ammonium citrate, 40 g l^−1^ MgSO_4_, 0.5 mg l^−1^ CaCl_2_, 0.6 mg l^−1^ ZnSO_4_, 0.6 mg l^−1^ CuSO_4_, 0.8 g l^−1^ NaCl, 0.5 g l^−1^ Tyloxapol, and 0.1% fatty acid-free bovine serum albumin (Sigma A8806) supplemented with 2 g l^−1^ of glucose or acetate. When required, 0.5 g l^−1^ of NH_4_SO_4_ was added as an alternative nitrogen source in lieu of glutamic acid. Bacterial growth was monitored by measuring culture OD_600_ (BioMate5 Fisher Scientific) at successive time points. All data for growth kinetics were derived from at least three independent experiments.

Viability of *M. smegmatis* and *M. bovis* BCG cultures was determined by plating serially diluted aliquots of cultures on LB solid medium for *M. smegmatis* or Middlebrook 7H9 solid medium supplemented with 0.5% glycerol and 10% oleic acid–albumin–dextrose–catalase (0.5 g l^−1^ oleic acid, 50 g l^−1^ bovine serum albumin fraction V, 20 g l^−1^ glucose, 40 mg l^−1^ catalase, 8.5 g l^−1^ NaCl) for *M. bovis* BCG. Colony-forming units (CFU) were scored after incubation of plates at 37 °C.

### Strain construction

Plasmids and bacterial strains are listed in Supplementary Table 4. All vectors used for mutant generation, complementation and conditional gene expression were constructed using TOPO Cloning Technology (Invitrogen). Unmarked in-frame gene deletions of *M. tuberculosis icd1* (*rv3339c*) and *M. smegmatis icd* (*msm1654*), *pknG* (*msm0786*), *icl1* (*msm0911*), and *icl2* (*msm3706*) were constructed using a method for two-step homologous recombination^44^. The in-frame recombination template was constructed by PCR amplification of ≈900 bp of the upstream and downstream region of the gene of interest. Primers were designed to introduce 5′ PacI and 3′ AvrII sites into the upstream amplicon including nine bases from the start codon, and 5′ AvrII and 3′ AscI into the downstream region including nine bases before the stop codon. The amplicons were verified by DNA sequencing then restriction digested and ligated into the PacI and AscI sites of pJG1100, a suicide vector that contains *aph* (kanamycin resistance), *hyg* (hygromycin resistance), and *sacB* (sucrose sensitivity) markers^45^. When inapplicable due to presence of internal restriction sites in the amplicons, NheI and SpeI were used instead to generate homologous arms.

Disruptions of the *M. bovis* BCG *icd1* (Mb3371c) and *icd2* (Mb0067c) genes were constructed by replacing the chromosomal locus with an interrupted (100 bp) in-frame *icd1::hyg* or *icd2::hyg* allele using a method for one-step homologous recombination^46^. Briefly, fragments of≈1 kb flanking the *icd1* or *icd2* open reading frame (ORF) were PCR amplified from *M. tuberculosis* H37Rv genomic DNA. The 5′- and 3′- flanking fragments were then cloned into pYUB854 flanking the hygromycin-resistance gene. A *sacB*-*lacZ* cassette excised from pGOAL17 (ref. 47) was then cloned into the unique PacI site of pYUB854. The final plasmids were ultraviolet-irradiated before electroporation into *M. bovis* BCG^48^. Positive clones of knockout mutants were selected as white colonies on 7H11 agar supplemented with 150 μg ml^−1^ hygromycin and 40 μg ml^−1^ X-Gal. Replacement of the target gene with the hygromycin-resistance marker was confirmed by PCR. Primers are listed in Supplementary Table 5.

Epitope-tagged translational fusions of *M. tuberculosis icd1 (rv3339c)*, *M. tuberculosis* *icd2 (rv0066c)*, *M. tuberculosis* *icl1 (rv0467)*, *M. tuberculosis* *icl2 (rv1916)* and *M. smegmatis* *icd (msm1654)* were constructed by inserting sequences encoding the epitopes VSVG, HA, FLAG, Myc or HA into the chromosome downstream of *rv3339c, rv0066c, rv0467, rv1916* or *msm1654* (respectively), just before the stop codon by two-step recombination with the suicide vector pJG1100. In all cases, the in-frame upstream recombination template was constructed by PCR amplification of ≈900 bp of the 3′ region of the gene of interest. Primers were designed analogous to that described for the unmarked gene deletion. The epitope tags were amplified with AvrII overhangs and were cloned into the AvrII site at the junction of the two homologous arms. The resultant plasmids were introduced into *M. smegmatis* and *M. tuberculosis* by electroporation. Transformants (first crossover) were selected on LB agar or 7H10 agar containing 50 μg ml^−1^ hygromycin and 25 μg ml^−1^ kanamycin. Individual colonies were picked and amplified in 7H9 liquid medium (no antibiotics) and then plated on LB agar or 7H10 containing 5% sucrose to select for cells in which plasmid excision had occurred (second crossover). Individual colonies were picked and the replacement of the gene by the fusion gene or gene deletion was confirmed by PCR. For *M. tuberculosis*, cells were plated on 7H10 agar in lieu of LB agar.

For complementation of the *M. bovis* BCG Δ*icd1*, *M. bovis* BCG Δ*icd2* or *M. smegmatis* Δ*icd* mutant, the corresponding ORF plus 200 bp of 5′-flanking sequence upstream of the translational start codon was PCR amplified and ligated into the *attB*-integrating promoter-less vector pMV306 digested with XbaI and HindIII to generate pMV306::P_np_*icd1*, pMV306::P_np_*icd2*, or pMV306::P_np_*icd*, respectively. Plasmids overexpressing the *M. bovis* BCG *icd1* or *icd2* gene were generated by PCR amplifying the respective ORFs and ligating into the BamHI and HindIII sites of pMV361 (a pMV306 derivative) downstream of the strong *hsp60* promoter to generate pMV306::P_hsp_*icd1* and pMV306::P_hsp_*icd2*. Plasmids were introduced into *M. bovis* BCG by electroporation and colonies were selected on 7H11 agar supplemented with 25 μg ml^−1^ kanamycin. Complemented strains were verified by PCR.

### Growth kinetics

Pre-cultures were inoculated from frozen stocks to an initial optical density at 600 nm (OD_600_)≈0.05. Cells were grown to early exponential phase (OD_600_≈0.5), collected by centrifugation (3,000*g*, 5 min), washed three times with phosphate-buffered saline (PBS) supplemented with 0.05% Tween-80 (Sigma), resuspended in fresh medium at OD_600_≈0.05, and incubated at 37 °C with aeration (180 r.p.m.). At specified time points, OD_600_ measurements were made using an Ultrospec 2000 spectrophotometer (Pharmacia). Where indicated, resuspended cells were seeded in microtiter plates, sealed with a gas-permeable adhesive seal (ThermoScientific, Waltham, USA), and incubated in a BioLector system^49^,^50^ for continuous monitoring of bacterial growth (OD_620_) and fluorescence.

### Time-lapse microscopy

Time-lapse microscopy of *E. coli* MG1655 (wild-type) and Δ*aceK* strains expressing YFP (Supplementary Table 4) was performed with minor modifications^51^. In brief, bacteria were grown to mid-log phase (OD_600_ ∼0.3) in M9 liquid medium containing 2 g l^−1^ glucose and 2 μl of culture were spread on a semipermeable membrane, which was then inverted onto a glass coverslip micro-patterned with rings of SU-8 (65 μm diameter × 1.5 μm height) containing a central post (10 μm diameter × 1.5 μm height). The bacteria-free side of the membrane was overlaid with a PDMS-based microfluidic device and the assembly was clamped between a transparent acrylic cover and base adapter, as described^51^. The inlet and outlet ports of the microfluidic device were connected to plastic tubing and bacteria were fed by continuous flow of M9 minimal medium containing 2 g l^−1^ glucose or acetate (flow rate, 25 μl min^−1^). Bacteria were maintained at 37 °C in a microscope-fitted environmental chamber and imaged using a DeltaVision personalDV microscope (Applied Precision) equipped with a 100 × oil-immersion objective. Images were recorded at 5-min intervals on phase-contrast and fluorescence channels (GFP, excitation filter 490/20/, emission filter 528/38) using a CoolSnap HQ2 camera. Images were processed using Softworx software (Applied Precision) and custom-made macros in ImageJ.

### Enzyme assays

ICL activity was measured using the following assay conditions: 25 mM imidazole buffer (pH 6.8), 10 mM EDTA, 5 mM MgCl_2_, 4 mM phenylhydrazine and 4 mM DL-isocitric acid trisodium salt hydrate. Enzyme assays were carried out in 96-well plates. Each well contained 100 μl of reaction master mix and 100 μl of purified recombinant protein (0.12 μg ml^−1^). Absorbance readings were taken at 324 nm using a temperature-controlled (25 °C) 96-well plate reader spectrophotometer (Tecan M200 Pro). ICL specific activity is defined as 1 U=1 μM phenylhydrazone glyoxylate generated per min (mg protein)^−1^.

ICD activity was measured by monitoring the time-dependent reduction of NADP^+^ to NADPH^14^ using the following assay conditions: 25 mM Tris–HCl pH 7.5, 5 mM MgCl_2_, 100 mM NaCl, 8 mM NADP^+^, and 8 mM DL-isocitric acid trisodium salt hydrate (Sigma). Enzyme assays were carried out in 96-well plates. Each well contained 100 μl of reaction master mix and 100 μl of purified recombinant protein (0.12 μg ml^−1^). Absorbance readings were taken at 340 nm using a temperature-controlled (25 °C) 96-well plate reader spectrophotometer (Tecan M200 Pro). ICD specific activity is defined as 1 U=1 μM of NADPH formed per min (mg protein)^−1^. ICD activity with varying glyoxylate concentrations was followed at 37 °C by flash-freezing the reactions at different times in 200 μl of acetonitrile. Samples were then prepared for mass spectrometry analysis to track the production of α-ketoglutarate. Stimulation of ICD activity by ICL was measured by following NADPH production at 340 nm in the presence of increasing amounts of recombinant wild-type ICL or mutated (catalytically inactive) ICL.

### Heterologous protein expression

ORFs encoding ICL and ICD were amplified by PCR. Amplicons were verified by DNA sequencing then restriction digested and ligated into a pET-28a(+) vector (Novagen USA) carrying a C-terminal 6-His tag and a TEV (tobacco etch virus) cleavage site preceding the C-terminal 6-His tag. The catalytically inactive ICL1^KKAGA^ was synthesized by GenScript, Inc. (Piscataway, NJ, USA) and similarly cloned into pET-28a(+) (Novagen USA) incorporating the C-terminal 6-His tag and TEV cleavage site. The final plasmids, designated pPM010 and pPM011 (catalytically active and catalytically, respectively), were transformed into chemically competent *E. coli* One Shot BL21 (DE3) (Invitrogen). Transformed bacteria were grown at 37 °C in LB liquid medium to early log phase (OD_600_ ∼0.3). Recombinant protein expression was induced by addition of 0.2 mM IPTG (isopropyl-β-D-thiogalactopyranoside) and bacteria were cultured at 16 °C for 24 h at 180 r.p.m. Cells were harvested by centrifugation (3,000*g*, 5 min), resuspended in lysis buffer (50 mM Tris pH 7.5, 500 mM NaCl, 1 mM imidazole, 10% glycerol, 1% Tween-20) containing DNase and mini-protease inhibitor cocktail (Roche), and incubated for 1 h at 4 °C. Cells were then disrupted by 20 cycles of sonication each comprising 15 s of power at 60% amplitude (Vibracell 75186, Bioblock Scientific) and 30 s of power off. Cell debris was pelleted by centrifugation at 30,000*g* for 30 min at 4 °C. The clarified supernatant was incubated with PrepEase resin (His-tagged protein purification resin-high specificity) for 12 h at 4 °C with gentle agitation (50 r.p.m.) and washed with 50 volumes of wash buffer (50 mM Tris pH 7.5, 500 mM NaCl, 5 mM imidazole). Proteins were eluted with 10 volumes of elution buffer (50 mM Tris–HCl pH 7.5, 500 mM NaCl, 250 mM imidazole). The purity of the eluent fractions was checked by SDS–PAGE and the 6-His tag was removed by cleavage with TEV (Promega). The native protein was purified by size-exclusion chromatography on Superdex 75 pg 10/300 GL columns (GE Healthcare). Fractions containing the eluted protein of interest were analyzed by SDS–PAGE to confirm purity (>98%, estimated by Coomassie Blue staining), concentrated using Vivaspin units (Sartiorius), and stored at −80 °C in buffer containing 50 mM Tris–HCl pH 7.5.

### Protein extraction and quantification

Cells were collected by centrifugation (3,500*g*, 15 min), and cell pellets were washed with Tris-buffered saline+0.02% tyloxapol, followed by a single wash with Tris-buffered saline alone at 4 °C. Washed cell pellets were resuspended in 50 mM Tris–HCl, pH 8.0, 10% glycerol, 1 × protease inhibitor (Roche Applied Science), 1 × phosphatase inhibitor (Pierce) and lysed at 4 °C in 15 s sonication cycles (Vibracell 75186, Bioblock Scientific) at amplitude 60% with 30 s intervals in-between cycles for 5 min. Finally, the cell debris was pelleted at 11,200*g*, 35 min, 4 °C and the protein concentration was estimated with a BCA protein quantification kit (ThermoScientific) with BSA standards prepared in the same buffer.

### Immunoblot analysis

Protein lysates were prepared in the same manner as described above. Between 50 and 100 μg total protein were separated on NuPAGE Novex 4–12% *bis*-Tris gels (Invitrogen) and transferred to nitrocellulose membranes (Bio-Rad, 162-0115, Hercules, CA, USA) for probing using the iBlot system (Invitrogen). Membranes were incubated in TBS blocking buffer (25 mM Tris–HCl pH 7.5, 150 mM NaCl, 0.05% Tween-20) with 5% w/v skim milk powder for 2 h at room temperature before incubation with primary antibodies (anti-FLAG (Clone M2, Sigma); anti-HA (Sigma); anti-Myc (Sigma); anti-VSVG (Clone 16B12, Sigma); anti-GroEL2, and anti-Ag85) diluted in TBS with 1% BSA overnight. All antibodies were used at a dilution of 1:1,000 except anti-GroEL2 (1:50,00) and anti-Ag85 (1:3,000). Membranes were washed five times in TBS (15 min each at room temperature) before incubating with appropriate secondary antibody for 1 h at room temperature. Membranes were developed using the colorimetric horseradish peroxidase substrate 4-chloro-1-naphthol (4CN) (Opit-4CN Kit by Bio-rad). Antibodies against GroEL2 and Ag85 were generous gifts from Dr J.M. Chen as part of the National Institutes of Health, National Institute of Allergy and Infectious Diseases, contract HHSN266200400091c entitled ‘Tuberculosis Vaccine Testing and Research Materials,' awarded to Colorado State University).

### X-ray crystallography

Crystals of *M. smegmatis* ICD were obtained by vapour diffusion at 18 °C by equilibrating 2 or 4 μl hanging drops containing a 1:1 mixture of 60 mg ml^−1^ protein pre-incubated with 10 mM glyoxylate, 10 mM MnCl_2_, 10 mM isocitrate, and 10 mM NADP^+^ in crystallization buffer containing 0.1 M Hepes pH 7.5, 0.05 M MgCl_2_, and 28–34% polyethylene glycol monomethyl ether 550 (Qiagen) over a 500 μl reservoir of the same crystallization solution. Crystals were stabilized by soaking briefly in a cryoprotectant solution (25% w/v glycerol in crystallization buffer) and flash frozen in liquid nitrogen before data collection. Diffraction data to 2.8 Å (ICD incubated with glyoxylate, isocitrate, MgCl_2_ and NADP) were collected on X06DA of the Swiss Light Source (SLS, PSI, Villigen, Switzerland). Data were indexed, integrated and scaled with XDS^52^. Phase determinations were carried out by molecular replacement using Phaser^47^ of the CCP4 Suite, using the published structure of AvICD (PDB: 1ITW) as a template. Structure figures were prepared with PyMOL (Molecular Graphics System, Version 1.5.0.1 Schrödinger, LLC). Crystallographic statistics are listed in Supplementary Table 1. Coordinates and structure factors have been deposited in the Protein Data Bank (PDB accession code PDB: 4ZDA).

### Molecular docking

*Protein preparation and grid generation*. The raw PDB structure of the ICD was converted into an all atom receptor model, fully prepared for docking studies, using Protein Preparation Wizard in Maestro (Maestro v.9.8). Default parameters in the preparation process were used. The protein structural integrity was checked and missing side chains were built. The protonation and tautomeric states of the residues were adjusted to match a pH of 7.4. Energy minimization of the whole structure was performed using an OPLS2005 force field. The docking receptor grid was created using Glide Receptor Grid Generation (Glide v.9.8). The grid box was centered between the two domains of ICD, in a region that allowed the grid box to include the whole protein structure. All the possible binding sites were explored.

*Ligand preparation*. The initial ligand file (sdf file, two-dimensional structure) was obtained from the Protein Data Bank (PDB). The glyoxylate was prepared for ligand docking using LigPrep from the Schrodinger suite. Default parameters in the ligand preparation process were used. A single low energy conformer with proper bond lengths and angles was generated from the two-dimensional structure. The protonation state was adjusted to match a pH of 7.4, tautomeric states were explored, and a final minimization step was performed. Glyoxylate was then docked inside the rigid 3D structure of the protein receptor (ICD), previously prepared. Glyoxylate was docked in the binding pocket and no other possible poses were found. An extra precision-docking revealed a binding energy of −6 kcal mol^−1^.

### Isothermal titration calorimetry (ITC)

ITC experiments were conducted on an automated micro-calorimeter. The experiments were performed at 25 °C in ITC buffer (10 mM Tris (pH 7.5), 10 mM EDTA, 50 mM NaCl_2_, 2.5 mM MgCl_2_). Protein concentrations were 15 mM for MsmICD, 150 mM glyoxylate (determined by weighing samples on an ultra-microbalance (Mettler Toledo, accurate to 0.001 mg). In titration experiments, glyoxylate was injected into a solution of MsmICD. In the competitive titration experiment, glyoxylate was injected into a solution of the enzyme. Titrations were carried out with a stirring speed of 750 r.p.m. and 300-s intervals between 4 μl injections. The first four injections were excluded from data fitting because effective mixing in the cell took longer than the injection intervals. Titrations were run past the point of enzyme saturation to correct for heats of dilution. The experimental data were fit to a theoretical titration curve using the Origin software package (version 7.0) provided with the instrument to afford values of *K*_A_ (the association constant in M^−1^), *n* (stoichiometry of binding), and Δ*H* (the binding enthalpy change in Kcal mol^−1^). ITC experiments were performed in duplicate, analyzed independently, and the obtained thermodynamic values were averaged.

### Metabolite measurements

Bacteria were grown in Middlebrook 7H9 liquid medium at 37 °C with aeration (180 r.p.m.) to mid-exponential phase, harvested by centrifugation (3,000*g*, 5 min) washed three times with PBS containing 0.05% Tween-80 (Sigma), inoculated into Middlebrook 7H9 liquid medium supplemented with 2 g l^−1^ glucose or acetate (initial OD_600_ 0.05), and grown at 37 °C to mid-log phase (OD_600_ ∼0.5). Samples equivalent to a biomass of 1 ml culture at OD_600_ 2 were collected for metabolome analysis by fast filtration and metabolism was quenched at –20 °C in a mixture of acetonitrile:methanol:water (2:2:1), as described^53^. Metabolites were measured by LC–MS/MS and direct injection, as described^25^,^54^. Glyoxylate was measured at 12.6±3.3 pmol ml^−1^ of acetate-grown culture at OD_600_ 1, which corresponds to a biomass of 343.4±17.5 μg. To estimate the intracellular concentration of glyoxylate in *M. smegmatis* we used a ratio of aqueous volume to cellular dry weight of 0.0023, l g^−1^, as determined for *E. coli*^55^.

### Data availability

Coordinates and structure factors have been deposited in the Protein Data Bank (PDB accession code PDB: 4ZDA). All additional data supporting the findings presented here are available from the corresponding authors upon request.

## Additional information

**How to cite this article:** Murima, P. *et al*. A rheostat mechanism governs the bifurcation of carbon flux in mycobacteria. *Nat. Commun.* 7:12527 doi: 10.1038/ncomms12527 (2016).

## Supplementary Material

Supplementary Information Supplementary Figures 1-8 and Supplementary Tables 1-5

Supplementary Movie 1 Time-lapse microscopy of GFP-expressing E. coli ΔaceK cells grown in a microfluidic device at 37°C and fed by continuous flow of M9 minimal medium containing glucose (0-3, 18-24, and 36-42 hours) or acetate (3-18 and 24-36 hours) as the sole source of carbon and energy. Images were recorded on the phase-contrast and fluorescence channels (merged) at 5-minute intervals using a 100X oil-immersion objective. The movie is a representative from one of at least three independent experiments. The carbon source is indicated in the top left corner. Scale bar, 3 μm.

## Figures and Tables

**Table 1:** ICD activity in cell-free extracts of bacteria grown on glucose or acetate.

**Bacterial strain**	ICD activity* **Glucose (Glc)**	ICD activity* **Acetate (Ace)**	**Ratio (Glc/Ace)**
*M. bovis* BCG	0.65±0.09	1.01±0.14	0.64
BCG Δ*icd1*	0.63±0.08	1.12±0.13	0.56
BCG Δ*icd2*	ND†	ND†	
BCG Δ*icd2 attB*::P_np_icd2‡	1.55±0.06	2.91±0.83	0.53
*M. smegmatis*	0.84±0.24	2.08±0.13	0.40
*M. smegmatis* Δ*icd2*	0.01±0.01	0.02±0.01	0.50
*M. smegmatis* Δ*icd attB*::P_np_icd†	1.02±0.18	3.19±0.42	0.32

Data are means±s.d. (*n*=3 independent experiments each performed in triplicate).

^*^ICD specific activity in cell-free extracts (U μg^−1^).

^†^Not detectable.

^‡^Strain complemented with single-copy pMV306-based plasmids integrated at the chromosomal *attB* site. In each case, the complementing gene is expressed from the native promoter (P_np_).

**Table 2:** ICD activity in cell-free extracts of bacteria grown on glucose or acetate.

**Bacterial strain**	ICD activity* **Glucose (Glc)**	ICD activity* **Acetate (Ace)**	**Ratio (Glc/Ace)**
*M. tuberculosis* Erdman	0.41±0.10	0.69±0.09	0.60
*M. tuberculosis* Erdman Δ*pknG*	0.46±0.14	0.62±0.03	0.75
*M. tuberculosis* H37Rv	0.42±0.04	0.63±0.08	0.66
*M. tuberculosis* CDC1551	0.35±0.03	0.58±0.06	0.61
*M. tuberculosis* HN787	0.44±0.08	0.62±0.03	0.72
*M. bovis* BCG	0.87±0.14	1.21±0.03	0.72
*M. smegmatis*	0.74±0.19	1.90±0.24	0.39
*M. smegmatis* Δ*pkn*G	0.71±0.21	1.36±0.06	0.52
*E. coli*	1.25±0.21	0.35±0.06	3.58
*E. coli* Δ*aceK*	1.09±0.14	1.18±0.08	0.93

Data are means±s.d. (*n*=3 independent experiments each performed in triplicate).

^*^ICD specific activity in cell-free extracts (U μg^−1^).

**Table 3:** ICL activity in cell-free extracts of bacteria grown on glucose or acetate.

**Bacterial strain**	ICL activity* **Glucose (Glc)**	ICL activity* **Acetate (Ace)**	**Ratio (Glc/Ace)**
*M. tuberculosis* Erdman	0.46±0.15	1.26±0.24	0.37
*M. bovis* BCG	0.36±0.04	0.84±0.08	0.43
*M. smegmatis*	0.22±0.03	1.76±0.26	0.13
*E. coli*	0.01±0.01	0.95±0.68	0.01

Data are means±s.d. (*n*=3 independent experiments each performed in triplicate).

^*^ICL specific activity in cell-free extracts (U μg^−1^).

**Table 4:** Thermodynamic parameters of glyoxylate binding to MsmICD.

**Compound**	***n***	***K*** **(M**^**−1**^**)**	**Δ*****H*** **(cal mol**^**−1**^**)**	**Δ*****S*** **(cal mol**^**−1**^**)**
Glyoxylate	1.17±0.02	1.10e^6^±4.21e^5^	1.129e^5^±4507	406
Succinate	0.12±12	3.05e^3^±1.34e^4^	–8.00e^4^±8.42e^6^	–252

Data are representative of three independent experiments with similar results.
