# Supplementary material for: A rheostat mechanism governs the bifurcation of carbon flux in mycobacteria
Source: Nat Commun. 2016 Aug 24;7:12527. doi: 10.1038/ncomms12527 (PMC4999502; doi:10.1038/ncomms12527)
Supplement: Supplementary Information — Supplementary Figures 1-8 and Supplementary Tables 1-5 [file ncomms12527-s1.pdf]

Supplementary Information

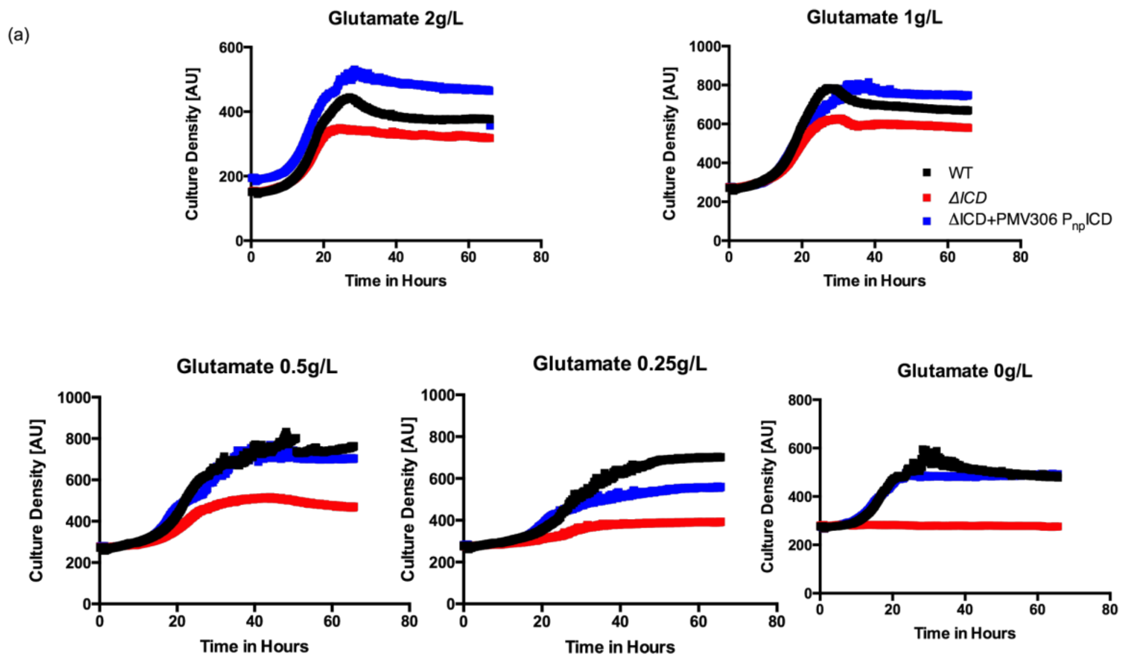

Supplementary Figure 1. Loss of ICD activity in *M. smegmatis* results in dose-dependent glutamate auxotrophy. Bacteria were grown in microtiter plates in minimal medium containing glucose as the sole carbon source plus glutamate at the indicated concentrations. Growth was monitored by taking continuous OD_{620} measurements with a BioLector system. Maximal growth of ICD-deficient bacteria declines with decreasing glutamate concentration in the medium, indicating that growth is impaired once glutamate is depleted. Data are representative of three independent experiments.

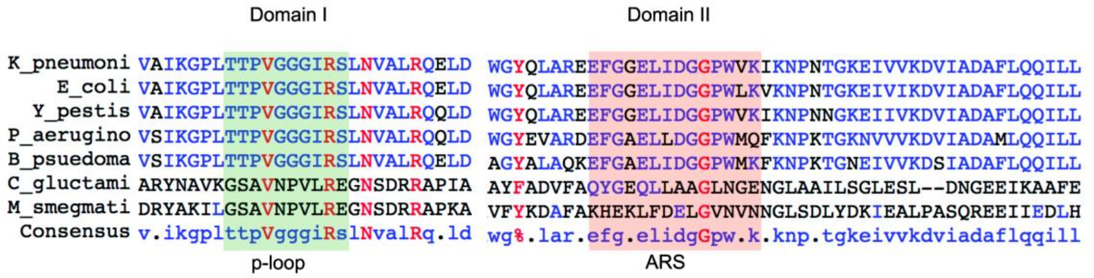

Supplementary Figure 2. Sequence-based alignment of a representative selection of ICD proteins from Gram-negative bacteria that also encode an AceK homolog. Conservation of the P-loop (green shading) and AceK Recognition Sequence (ARS, red shading) regions is evident.

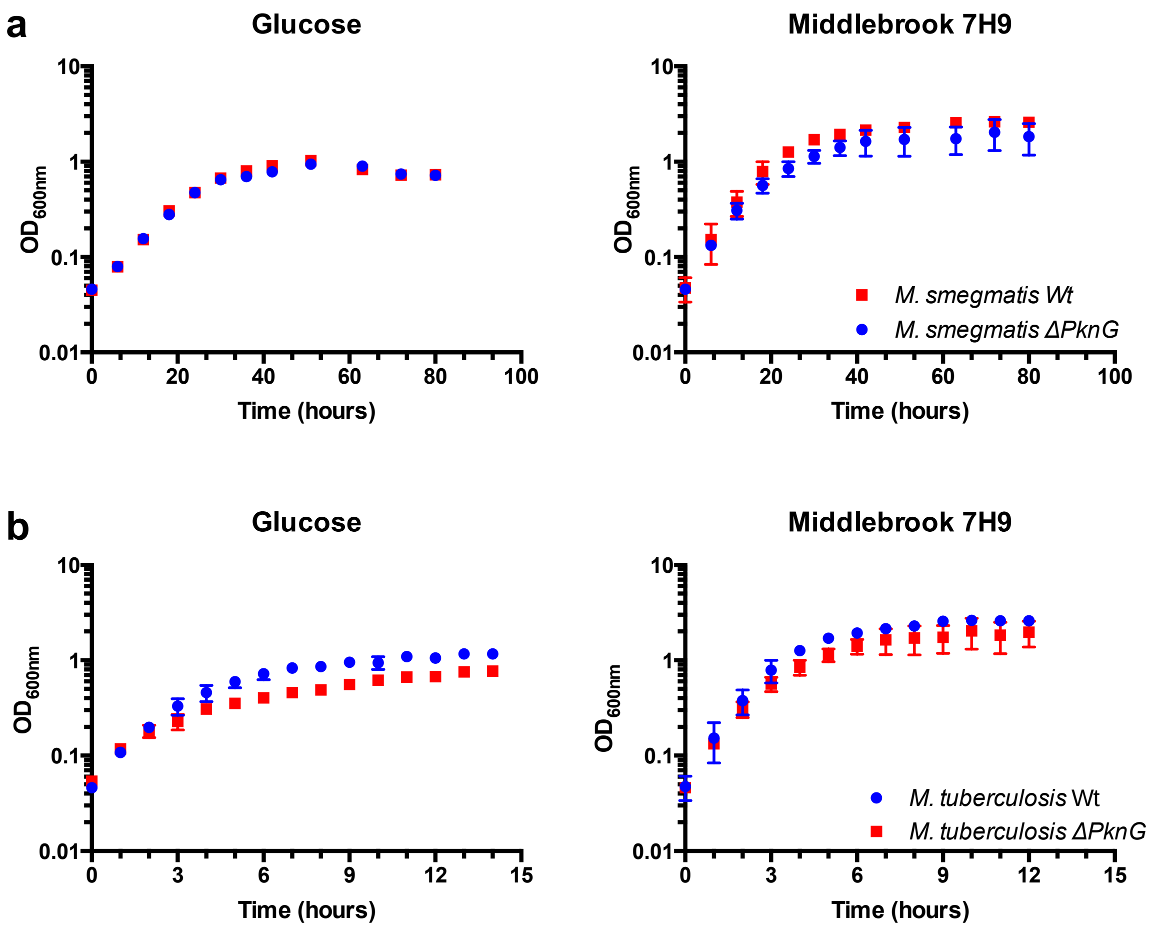

Supplementary Figure 3. PknG deficiency does not affect *in vitro* growth of *M. smegmatis* (upper panels) or *M. tuberculosis* (lower panels) in minimal medium with glucose as the carbon/energy source (left panels) or nutrient-rich medium with glucose as the carbon/energy source (right panels).

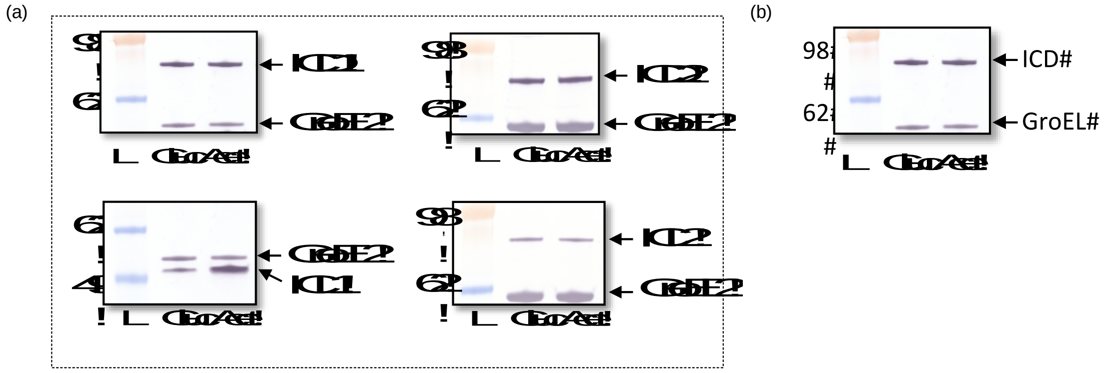

Supplementary Figure 4. ICD isozymes are not differentially expressed in minimal media with limiting glucose (Gluc) or acetate (Acet) as the sole carbon/energy source.

(a) Immunoblots of protein extracts from *M. tuberculosis* expressing HA-tagged ICD2, VSVG-tagged ICD1, FLAG-tagged ICL1, and Myc-tagged ICL2.

(b) Immunoblots of protein extracts from *M. smegmatis* expressing HA-tagged ICD.

L, molecular size marker (kDa). GroEL, loading control.

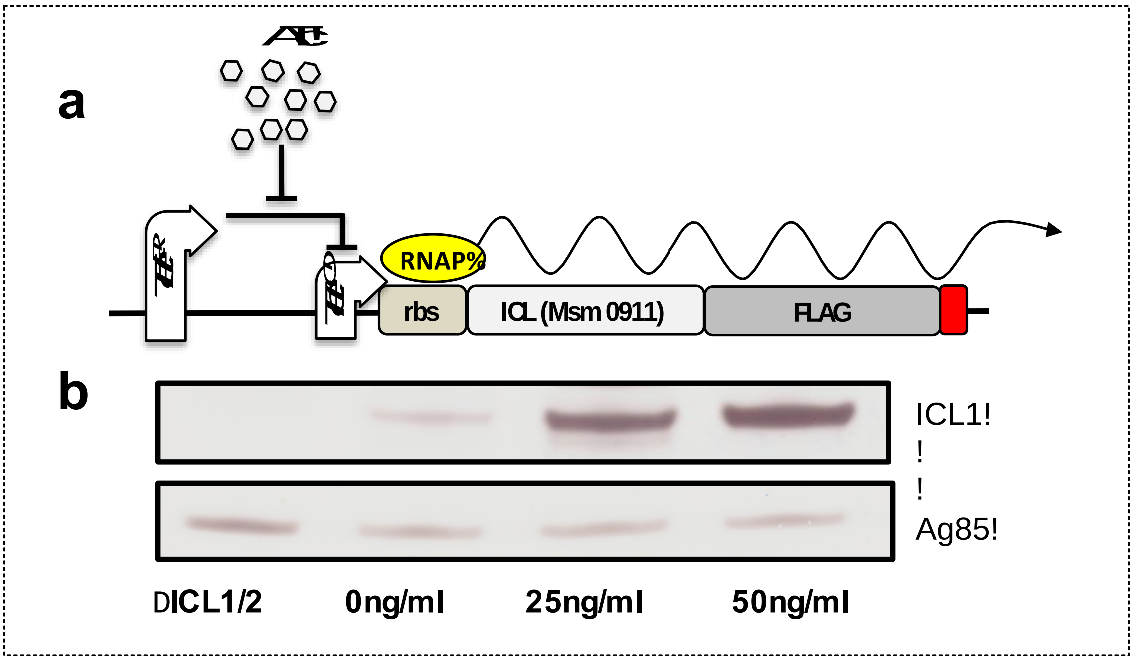

Supplementary Figure 5. Conditional expression of FLAG-tagged ICL1.

(a) Schematic of the *M. smegmatis icl1-flag* fusion gene expressed from the ATc-inducible Tet^{On} promoter. The construct was integrated at the lone *attB* site in the chromosome of an Δ*icl1* Δ*icl2* double-deletion strain.

(b) Immunoblots of protein extracts of the *M. smegmatis* Δ*icl1* Δ*icl2 icl1-flag* strain cultured at different inducer (ATc) concentrations. Protein extracts were prepared from cultures grown for 18 hours in minimal medium with glucose as the sole carbon source plus ATc at the indicated concentration. Upper panel, anti-FLAG antibody; lower panel, anti-Ag85 antibody (loading control). Data are representative of at least two independent experiments.

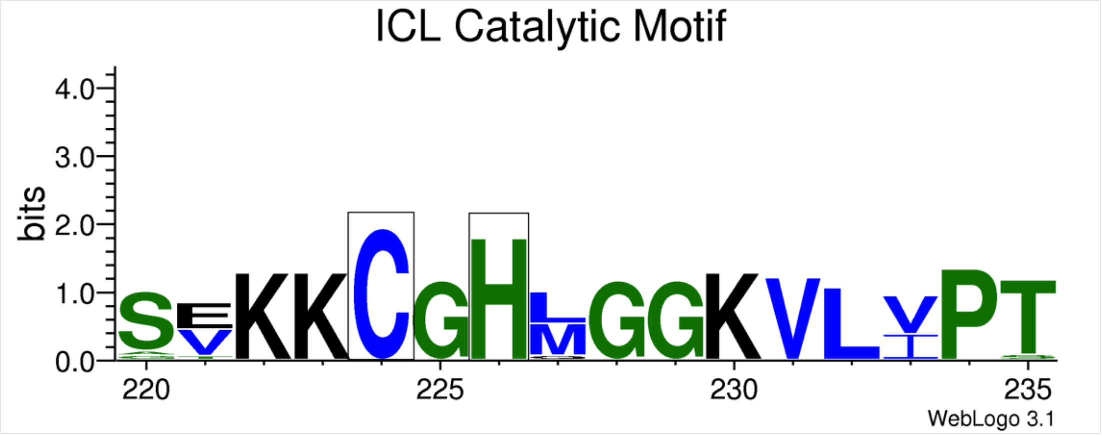

Supplementary Figure 6. Sequence conservation of the ICL catalytic motif. The ICL signature catalytic motif KKCGH has the two most conserved residues from 20 representative bacterial genomes encoding an ICL homolog. The boxed amino acid residues (C224A and H226A) were mutated to generate a catalytically inactive *M. smegmatis* ICL1 protein.

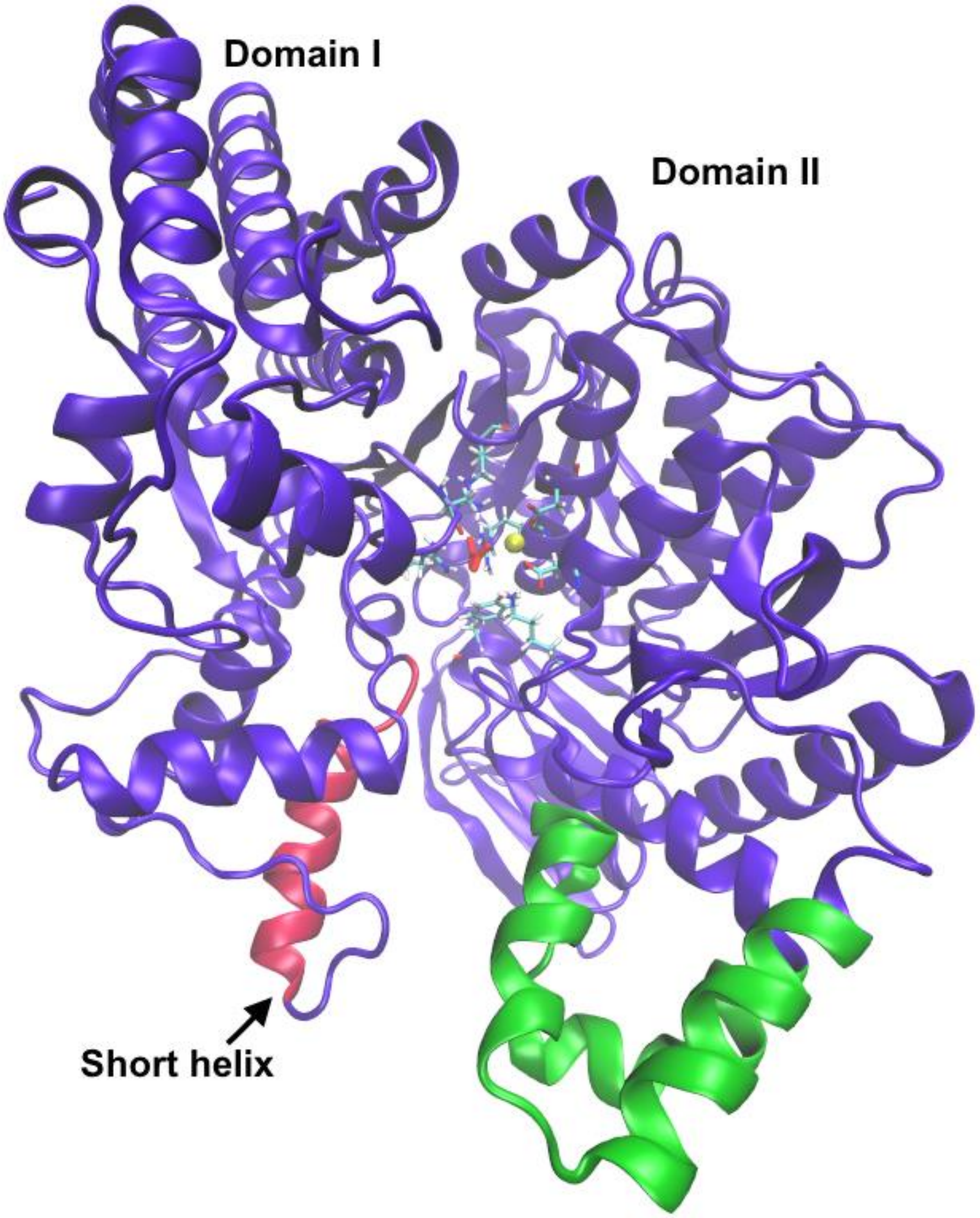

Supplementary Figure 7. View of putative glyoxylate binding site located between the large domain (II) and small domain (I) of the MsmICD protein. The P-loop domain is colored in red. The ARS domain is colored in green.

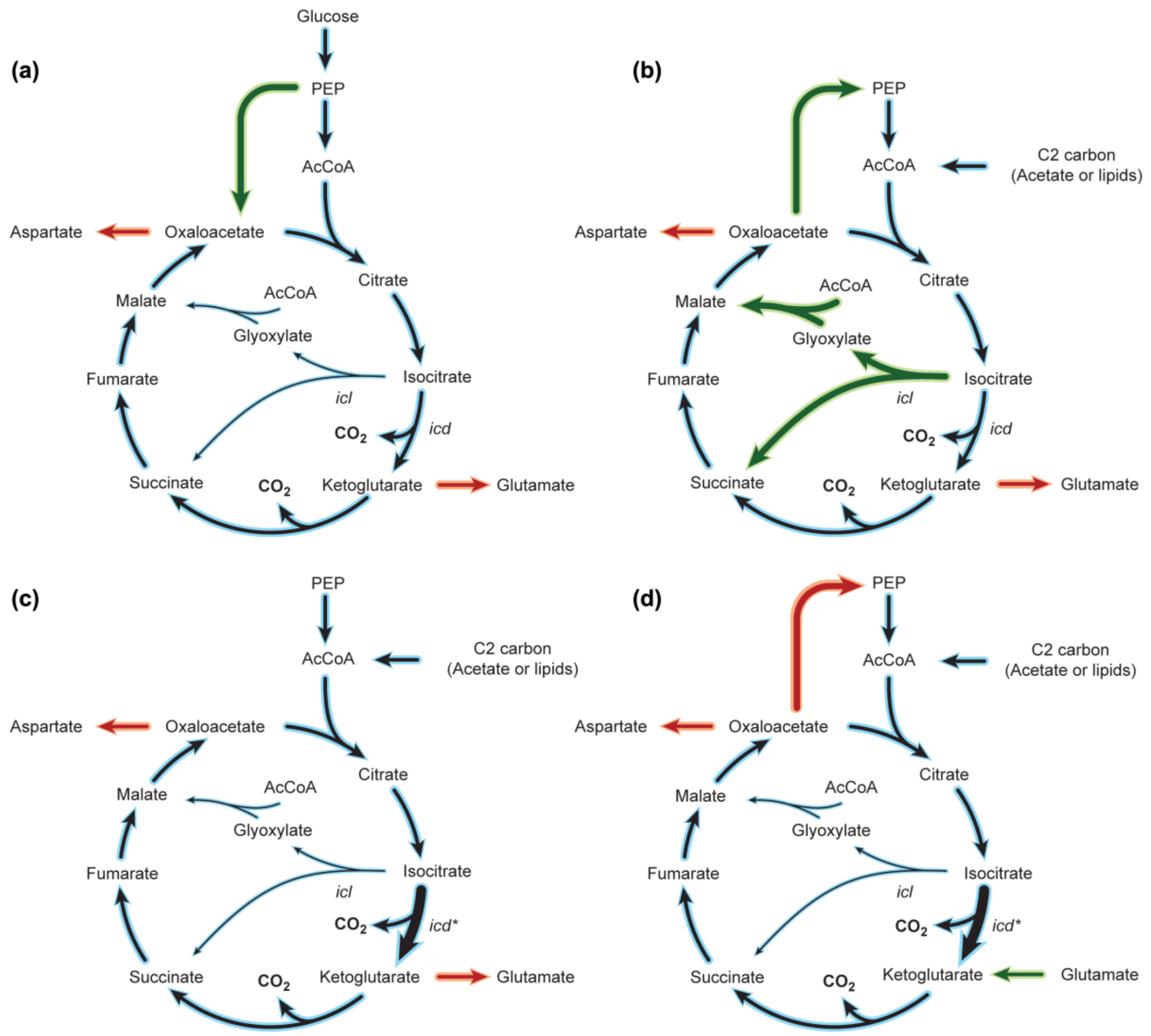

Supplementary Figure 8. Metabolic consequences of deregulating flux bifurcation between the oxidative TCA cycle and the glyoxylate shunt during growth on glucose (a), on acetate with glutamate (b, c), or on acetate without glutamate (d). Green arrows denote condition-dependent anaplerotic pathways. Red arrows denote carbon efflux from the TCA cycle. Thickness of arrows indicates putative fluxes. ICD* indicates overexpression of ICD (c,d).

(a) Carbon anaplerosis is mediated by PEP carboxylase during growth on glucose.

(b), Carbon anaplerosis is mediated by the glyoxylate shunt during growth on acetate.

(c) In cells with imbalanced flux partitioning due to ICD overexpression (ICD*), growth on acetate is impaired due to insufficient anaplerotic flux through the glyoxylate shunt.

(d) In cells with imbalanced flux partitioning due to ICD overexpression (ICD*), growth on acetate can be restored by addition of glutamate to the culture medium. Conversion of glutamate to α-ketoglutarate allows replenishment of TCA cycle intermediates, thus bypassing the anaplerotic function of the glyoxylate shunt.

Supplementary Table 1: Effects of ICD gene deletion in the tested mycobacterial species.

Strain	Gene deletion	Phenotype
M. tuberculosis	ICD1 (Rv3339c)	No growth defect. ICD specific activity is equivalent to wild-type.
M. tuberculosis	ICD2 (Rv0066c)	N.D
M. bovis BCG	ICD1 (Mb3371c)	No growth defect. ICD specific activity is equivalent to wild-type.
M. bovis BCG	ICD2 (Mb0067c)	Auxotrophic for glutamate. ICD specific activity is undetectable.
M. smegmatis	ICD (Msmeg_1654)	Auxotrophic for glutamate. ICD specific activity is undetectable.

N.D., Not Done. Multiple attempts to delete ICD2 (Rv0066c) in *M. tuberculosis* were unsuccessful.

Supplementary Table 2. Crystallographic data and refinement statistics for *M. smegmatis* ICD (MsmICD). Molecular replacement using the monomeric ICD of *Azotobacter vinelandii* (AvICD, PDB: 1ITW) as template.

**PDB Code**	**4PCX**[1]
**Space Group**	C2
**Cell Dimensions: *a, b, c* (Å)**	201.77, 206.16, 145.60
**Cell Dimensions: *α, β, γ* (°)**	90.00, 90.97, 90.00
**Resolution (Å)**	50.00 (2.80)
R_{meas}	61.9 (7.1)
$I / \sigma I$	12.81 (1.81)
**Completeness (%)**	97.4 (93.9)
**Redundancy**	2.16 (2.18)
**Resolution (Å)**	2.80
**No. Reflections**	138134
R_{work} / R_{free}	0.21 / 0.26
**No. Atoms: Proteins**	34483
**No. Atoms: Isocitrate/Mn^{2+}**	78 / 6
***B*-factors ($Å^2$)**	68.00
**R.m.s. Deviations: Bond Lengths**	0.005
**R.m.s. Deviations: Bond angles (°)**	1.033
**Ramachandran Plot:**	
**Preferred/Allowed Regions (%)**	99.59
**Outliers (%)**	0.41

[1]Highest resolution shell is shown in parentheses.

Supplementary Table 3. Overexpression of ICD1 or ICD2 in *M. bovis* BCG.

Data are means ± SD (n ≥ 3 independent experiments each performed in triplicate).

Strain	ICD Activity[1]	Fold Change[2]
M. bovis BCG (wild-type)	0.68±0.05	1
BCG *attB*::pMV306 (empty vector)	0.61±0.08	0.89
BCG *attB*::P_{np}icd1[3]	0.93±0.12	1.4
BCG *attB*::P_{np}icd2[3]	0.87±0.09	1.3
BCG *attB*::P_{hsp}icd1[3]	22±1	33
BCG *attB*::P_{hsp}icd2[3]	1.6±0.08	2.3

[1]ICD specific activity in cell-free extracts (U μg^{-1}).

[2]Relative to the wild-type strain.

[3]Strain carries a single-copy pMV306-based plasmid integrated at the chromosomal *attB* site. The encoded gene (*icd1* or *icd2*) is expressed from the native promoter (P_{np}) or overexpressed from the *hsp60* promoter (P_{hsp}).

Supplementary Table 4a. Plasmids used in this study.

**Plasmid Name**	**Marker**	**Description**	**Source**
pMV306	Km^R	Single-copy *attB*-integrating vector	Dr. W.R. Jacobs
pMV306::P_{np}icd1	Km^R	pMV306 expressing *icd1* (*rv3339c*) from the native promoter	This study
pMV306::P_{hsp}icd1	Km^R	pMV306 expressing *icd1* (*rv3339c*) from the *hsp60* promoter	This study
pMV306::P_{np}icd2 (RV0066c)	Km^R	pMV306 expressing *icd2* (*rv0066c*) from the native promoter	This study
pMV306 P_{np} ICD (MSM1654)	Km^R	pMV306 expressing ICD (*msm1654*) from the native promoter	This study
pND255	Hyg^R	Tet^{on} vector	Dr. N. Dhar
pPM004::icl1	Hyg^R	Tetracycline-induced (Tet^{on}) expression of *icl1* (*msm0911*) with an HA Epitope-tag	This study
pET28a(+)	Amp^R	Plasmid for production of recombinant proteins in *E. coli* BL21 (DE3).	Novagen
pPM001::icl1	Amp^R	pET28a(+) encoding ICL1 (*msm0911*)	This study
pPM010::icd	Amp^R	pET28a(+) encoding ICD (*msm1654*)	This study
pPM011::$icl1^{KKAGA}$	Amp^R	pET28a(+) encoding $ICL1^{KKAGA}$ (*msm0911*) mutated in active-site KKCGH motif (C224A and H226A)	This study

Supplementary Table 4b. Bacterial strains used in this study.

**Strain Name**	**Description**	**Source**
M. tuberculosis H37Rv	Lab strain	ATCC # 27294
M. tuberculosis CDC1551	Clinical isolate	Dr. P. Bifani
M. tuberculosis HN787	Clinical isolate	Dr. P. Bifani
M. tuberculosis Erdman	Lab strain (wild-type)	Lab collection
M. tuberculosis Δ*icd1*	In-frame unmarked deletion of *icd1*	This study
M. tuberculosis Δ*pknG*	In-frame unmarked deletion of *pknG*	This study
M. tuberculosis TCT3	HA-ICD2, VSVG-ICD1, FLAG- ICL1, and Myc-ICL2	This study
M. bovis BCG	Vaccine strain (wild-type)	Pasteur # 1173P2
M. bovis BCG Δ*icd1*	Δ*icd1*::hyg^R null mutant	This study
M. bovis BCG Δ*icd2*	Δ*icd2*::hyg^R null mutant	This study
M. smegmatis mc^2155	Lab strain (wild-type)	ATCC # 700084
M. smegmatis Δ*icd*	In-frame unmarked deletion of *icd*	This study
M. smegmatis Δ*pknG*	In-frame unmarked deletion of *pknG*	This study
M. smegmatis Δ*icl1* Δ*icl2*	In-frame unmarked deletions of *icl1* and *icl2*	Dr. M. Lotlikar
M. smegmatis TCS3	Epitope-tagged ICL1-FLAG, ICL2-Myc, and ICD-HA	This study
E. coli MG1655	Lab strain (wild-type)	Lab collection
E. coli Δ*aceK*	*aceK* null mutant	Yale E. coli Genetic Stock Center. CGSC# 10859

Supplementary Table 5. Primers used in this study.

Primer Name	Forward (5'-3')	Reverse (5'-3')
ICD1_Hsp	ATGGATCCATGTCCAACGCACC	CGATGAAGCTGGTGATCAAGCTTAT
ICD2_Hsp	ATGGATCCATGAGCGCCGAACAG	GATGTCACCGAACGAGTTTGATTAAGCTTAT
ICD1_Np	ATTCTAGAGCTGTCGGCGCTCATGAGCTT	AGCACTCTTCGATGAAGCTGGTAAGCTTAT
ICD2_Np	ATTCTAGAGCGATGCAGAGCTACAACGCT	GATGTCACCGAACGAGTTTGATTAAGCTTAT
MsmICD_Hsp	ATGGATCCATGAGCGCCCAGCAGCCGAC	AATCTCGGCGTCCTAGGCGTCAGCAAGCTTAT
MsmICD_Np	ATTCTAGAAAGAGACTATCCCACCGCTCACAG	AATCTCGGCGTCCTAGGCGTCAGCAAGCTTAT
BCG Δ*icd1*_Up	ATCTTAAGTGGTGGCCGAATGCACGACGA	AAGCTCATCAAGGACATGCTTATTCTAGAAT
BCG Δ*icd1*_Dw	ATAAGCTTAGATGACCAAGGACCTCGCGAT	ACTGCGCATAGAGCCGACACTAACTAGTAT
BCG Δ*icd2*_Up	ATCTTAAGTCTCGAATCCAAGTCCGGTGTT	TTCTGCCGATTGTGCGTGCCTTTCTAGAAT
BCG Δ*icd2*_Dw	ATAAGCTTAGACGTCATCGTGCGAGAGCT	TGGCGCGCTGGCGGCATCAACTAGTAT
Msm Δ*icd*_Up	ATTTAATTAATGTTGCCGGGAACCTCAAG	GGAGAGGAACCATGAGCACTAGTAT
Msm Δ*icd*_Dw	ATACTAGTTCGGTGTAACCCGATCC	AGCGAGTTGCCGACGATGTGGCGCGCCTT
Msm Δ*pknG*_Up	ATTTAATTAACACCGGGTTCGACGTCGA	GTACGTCGACTGATGACTCCTAGGAT
Msm Δ*pknG*_Dw	AACCTAGGACGTTCTAGCTCAACCCA	TTCGCCGACGAATCGCGGAGTCGAGCTCAG
Mtb Δ*icd1*_Up	ATTTAATTAACAGGTTCAAGATGGCGAACG	CTCCCATGTCCAACAGCTAGCAT
Mtb Δ*icd1*_Dw	AAGCTAGCTCGCCGACAACCTGGAAAAGGAGCTGGCCAATTA	ACGAGTTGCCGACGATATGGCTGGTTTGGCGCGCCAT
Mtb Δ*pknG*_Up	ATTTAATTAACGGCTTCGACGTTGACATCGC	GGTATGTGGACTGATGGCCCCTAGGTA
Mtb Δ*pknG*_Dw	ATCCTAGGACGTTCTAAGCCGCCCGAGTG	ATCTGATTCAAACCGTCGAGCGGGGGCGCGCCAT
Msm_ICL_Tet^{On}	AAGCTAGCATGTCGACCGTTGGCACCCCGAAG	GGGACTACAAGGACGATGACGACAAGTAAAGTACTTT
Msm_rICD[#]	ATCCATGGATGAGCGCCCAGCAGCCGACCATC	TTCAACACCACGCTCGAATCGGTGAAGCTTAT
Msm_rICL[#]	ATCCATGGTGTCTGTCGTCGGCACCCCGAA	ACCGAAGAGGGCCAGTTCCACAGGAAGCTTAT

*Restriction sites are underlined. Extra bases (AA/AT) were added at the end of the DNA strands for efficient cleavage. [#]The stop codons were removed to enable in-frame fusion with six histidine residues encoded in the vector.
